# Preferential Co substitution on Ni sites in Ni–Fe oxide arrays enabling large-current-density alkaline oxygen evolution†

**DOI:** 10.1039/d2sc02019j

**Published:** 2022-05-31

**Authors:** Yuping Lin, Xiaoming Fan, Mengqiu Huang, Zeheng Yang, Weixin Zhang

**Affiliations:** School of Chemistry and Chemical Engineering, Anhui Key Laboratory of Controllable Chemical Reaction and Material Chemical Engineering, Hefei University of Technology Hefei 230009 PR China xmfan@hfut.edu.cn wxzhang@hfut.edu.cn; Institute of Energy, Hefei Comprehensive National Science Center Anhui Hefei 230009 PR China

## Abstract

Developing low-cost and high-activity transition metal oxide electrocatalysts for an efficient oxygen evolution reaction (OER) at a large current density is highly demanded for industrial application and remains a big challenge. Herein, we report vertically aligned cobalt doped Ni–Fe based oxide (Co–NiO/Fe_2_O_3_) arrays as a robust OER electrocatalyst *via* a simple method combining hydrothermal reaction with heat treatment. Density functional theory calculation and XRD Rietveld refinement reveal that Co preferentially occupies the Ni sites compared to Fe in the Ni–Fe based oxides. The electronic structures of the Co–NiO/Fe_2_O_3_ could be further optimized, leading to the improvement of the intrinsic electronic conductivity and d-band center energy level and the decrease in the reaction energy barrier of the rate-determining step for the OER, thus accelerating its OER electrocatalytic activity. The Co–NiO/Fe_2_O_3_ nanosheet arrays display state-of-the-art OER activities at a large current density for industrial demands among Fe–Co–Ni based oxide electrocatalysts, which only require an ultra-low overpotential of 230 mV at a high current density of 500 mA cm^−2^, and exhibit superb durability at 500 mA cm^−2^ for at least 300 h without obvious degradation. The Co–NiO/Fe_2_O_3_ nanosheet arrays also have a small Tafel slope of 33.9 mV dec^−1^, demonstrating fast reaction kinetics. This work affords a simple and effective method to design and construct transition metal oxide based electrocatalysts for efficient water oxidation.

## Abstract 2

Co–NiO/Fe_2_O_3_ nanosheets featuring Co substitution on Ni sites can effectively regulate electronic structures and exhibit high OER activities with low overpotential (*η*_500_ = 230 mV), small Tafel slope (33.9 mV dec^−1^) and superb durability for 300 h.

## Graphical abstract

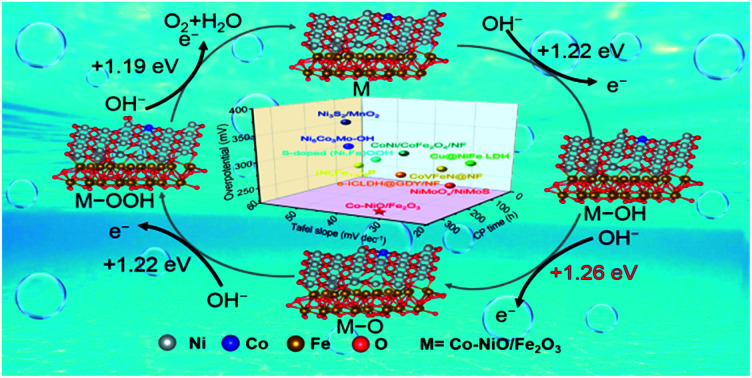

## Introduction

The oxygen evolution reaction (OER) involves a four-electron transfer process with slow reaction kinetics,^1,2^ which has been considered as an important half-reaction for water splitting, CO_2_/N_2_ electroreduction, and metal–air batteries, and significantly affects the overall reaction efficiency.^3–6^ Tremendous efforts have been devoted to exploring catalysts with high activity toward the OER *via* decreasing the anodic overpotential.^7,8^ So far, Ir/Ru based alloys and oxides have expressed excellent OER electrocatalytic activity in alkaline media, while the high cost and scarcity restrict their industrial applications.^9–11^ Therefore, rational design and facile synthesis of cost-effective and earth-abundant OER electrocatalysts with superb activity and stability remain a challenge.

Transition metal-based electrocatalysts, especially transition metals such as Fe, Co, and Ni with abundant resources and controllable electronic and crystal structures, have been recognized as the most promising candidates to replace noble metals.^12–14^ Recently, a series of Fe, Co, and Ni-based compounds, including oxides, (oxy)hydroxides, phosphides, borides, carbides, nitrides, sulfides, and selenides, have been reported as active OER electrocatalysts.^15–20^ Among them, Fe, Co, and Ni-based oxides present better prospects for the industrial application due to their feasible scaled-up production and higher thermodynamical stability. However, these oxide electrocatalysts still suffer from low energy conversion efficiency due to their poor electronic conductivity resulting from their intrinsic semiconductor characteristic and inappropriate adsorption energy for OER intermediates, which cause a high overpotential for oxygen evolution, especially at large current densities for the industrial requirement (>500 mA cm^−2^ with an overpotential lower than 300 mV).^21,22^

Currently, various strategies have been developed to provide Fe, Co, and Ni-based oxides with high electrocatalytic activities comparable to noble metals or other types of transition metal-based electrocatalysts.^23–26^ Doping metal or non-metal elements is adopted as an effective strategy to enhance the OER activity of Fe, Co, and Ni-based electrocatalysts because the electronic structures of electrocatalysts could be tuned to enhance the intrinsic electronic conductivity and optimize the adsorption/desorption energy of active species in the OER process.^27,28^ Wu *et al.* reported that the electron transfer and hydrogen/water adsorption free energy were significantly improved after the incorporation of Fe cations into Ni_3_S_2_, and the obtained Fe_0.9_Ni_2.1_S_2_ @ NF catalysts required an overpotential of 252 mV at 100 mA cm^−2^ for the OER in 1.0 M KOH.^29^ Mai *et al.* demonstrated that the OER activity of phosphorus doped Co_3_O_4_ was effectively enhanced due to the coupled P–O groups which promoted the metal–oxygen covalency and accelerated the electron transfer between the active metallic center and oxygen adsorbates.^30^ The formation of binary or ternary mixed metal oxides is also an effective way to modulate the electronic structures. Gao *et al.* successfully synthesized self-supporting NiO/Co_3_O_4_ hybrids with abundant heterointerfaces and oxygen vacancies at the interfaces, which resulted in the generation of numerous low-coordination atoms, and the d electrons of Co were regulated effectively. The catalytic activity of NiO/Co_3_O_4_ heterostructures was greatly enhanced with a low overpotential of only 262 mV at 10 mA cm^−2^.^31^ However, Fe, Co, and Ni-based oxides still face tremendous challenges towards meeting the industrial demands for stable OER electrocatalysts with low overpotentials at large current densities.

Herein, we present NiO/Fe_2_O_3_ oxide nanosheet arrays featuring preferential Co substitution on Ni sites (denoted as Co–NiO/Fe_2_O_3_), which were used as an OER electrocatalyst and showed high electrocatalytic performance with low overpotentials, fast reaction kinetics and high stability. Co–NiO/Fe_2_O_3_ nanosheet arrays were directly grown on a Ni foam substrate by a simple hydrothermal reaction and subsequent heat treatment. Density functional theory (DFT) calculation and characterization results proved that Ni was more preferentially substituted by Co in the NiO/Fe_2_O_3_ oxides, leading to the regulation of the electronic structure of Co–NiO/Fe_2_O_3_, and the reduction of the energy barriers for the rate-determining step in the OER process. Notably, the Co–NiO/Fe_2_O_3_ nanosheet arrays exhibited a low overpotential of only 230 mV at a high current density of 500 mA cm^−2^ and a small Tafel slope of 33.9 mV dec^−1^, and demonstrated superb durability for at least 300 h without significant degradation at 500 mA cm^−2^. This study would provide an effective strategy to regulate the electronic structures of transition metal oxides as advanced electrocatalysts for oxygen evolution.

## Results and discussion

### Fabrication and characterization of catalysts

**Fig. 1:**
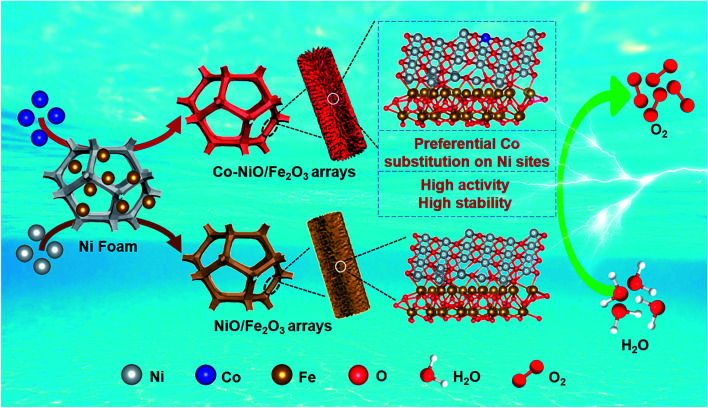
Schematic illustration of the preparation of Co–NiO/Fe_2_O_3_ nanosheet arrays.

To fabricate Co–NiO/Fe_2_O_3_ nanosheet arrays, the precursors containing Fe, Co, and Ni were produced by a simple hydrothermal reaction first, in which Ni foam was used as the substrate and nickel source as well. As shown in Fig. 1, the above hydrothermal reaction system only consists of Ni foam and a mixed aqueous solution of Co(NO_3_)_2_·6H_2_O and FeSO_4_·7H_2_O, and no generally required controlling reagents (*e.g.* urea, NH_4_F) were added.^32–34^ The precursors composed of 3Ni(OH)_2_·2H_2_O and FeOOH are formed after the hydrothermal reaction involving both Co^2+^ and Fe^2+^ (Fig. S1a†). The formation of FeOOH may be due to that the hydrolysis product Fe(OH)_2_ could be easily oxidized to FeOOH by the dissolved oxygen. However, the corresponding diffraction peaks related to Co element could not be observed in the XRD pattern. Nevertheless, the Co signals could be detected in the XPS spectra, suggesting the presence of Co in the Fe–Co–Ni based precursors (Fig. S1c†). Meanwhile, we find the importance of Co^2+^ in the system which triggers the co-deposition of the precursors containing Fe, Co, and Ni. In contrast, no nanosheet arrays or other nanostructures could be observed on Ni foam when the system only contains Fe^2+^ and Ni foam (Fig. S2†). Moreover, only the diffraction peaks of the Ni foam substrate could be observed in the XRD pattern, and the survey and Fe/Ni 2p XPS spectra confirm the existence of Ni and O elements while no Fe signals could be detected (Fig. S3†). To further understand the growth mechanism of the precursors, the changes of pH values during the hydrothermal reaction were measured (Fig. S4†). The initial pH value of Co(NO_3_)_2_ and FeSO_4_ mixed solution is 4.91, revealing an acidic reaction environment in the initial stage. The pH value then decreases rapidly and reaches 3.44 after 3 h, which is mainly due to the generation of H^+^ ions from the hydrolysis of Co^2+^ ions (stage I). With increase in the reaction time, Fe^2+^ ions begin to hydrolyze in such an acidic solution, and the as-generated H^+^ ions further reduce the pH values of the system (stage II). Meanwhile, the H^+^ ions derived from the hydrolysis of Co^2+^ and Fe^2+^ etch the Ni foam substrate to produce Ni^2+^.^35^ In the last stage of this reaction, the pH value of the solution increases slowly which could be attributed to the co-deposition reaction of Fe^2+^, Co^2+^, and Ni^2+^ to form the Fe–Co–Ni based precursors (stage III). Accordingly, we propose that the hydrolysis of Co^2+^ in the system could provide a suitable acidic solution to trigger the hydrolysis of Fe^2+^ and subsequent co-deposition of the precursors containing Fe, Co, and Ni, demonstrating the important role of Co^2+^ in the system. Finally, the above precursors were calcined at 450 °C for 2 h to produce Co–NiO/Fe_2_O_3_ nanosheet arrays. For comparison, NiO/Fe_2_O_3_ nanosheet arrays were prepared in a similar way in which additional Ni^2+^ instead of Co^2+^ was added in the hydrothermal reaction.

**Fig. 2:**
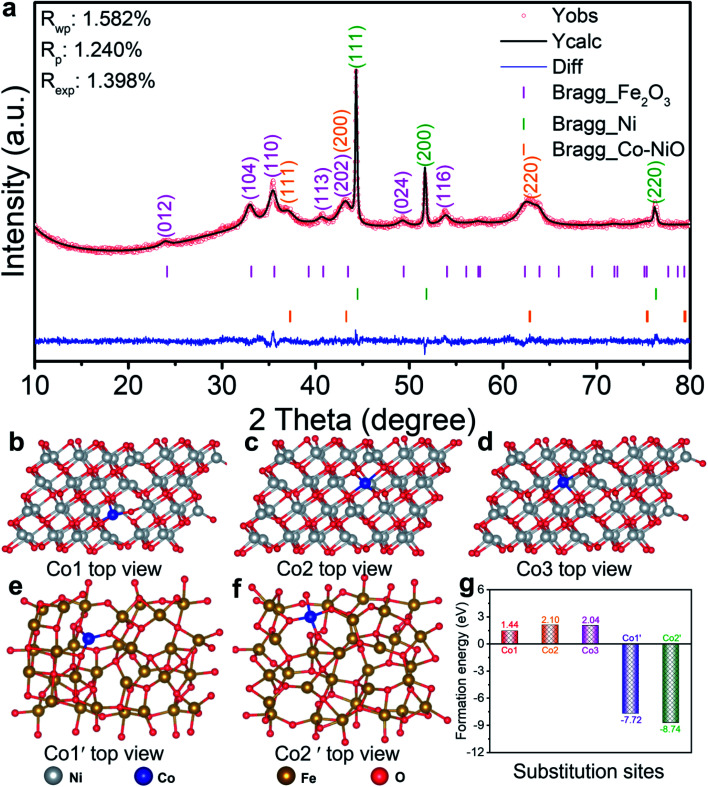
(a) Rietveld refined XRD pattern of the Co–NiO/Fe_2_O_3_ with partial Ni sites substituted by Co. Top views of (b) Co1, (c) Co2 and (d) Co3 substitution sites on NiO, and (e) Co1′ and (f) Co2′ substitution sites on Fe_2_O_3_. (g) The formation energy of Co substitution on different sites in NiO and Fe_2_O_3_.

Co–NiO/Fe_2_O_3_ nanosheet arrays were peeled from the Ni foam substrate to characterize the phase and composition by powder X-ray diffraction (XRD) measurement (Fig. 2a). The peaks at 37.2°, 43.3° and 62.8° can be attributed to cubic NiO (PDF no 71-1179), and the peaks around 24.2°, 33.2°, 35.6°, 40.9°, 43.5°, 49.5°, 54.1°, 62.4° and 64.0° can be ascribed to hexagonal Fe_2_O_3_ (PDF no 84-0306). In addition, the obvious peaks (44.5°, 51.8° and 76.4°) of metallic cubic phase Ni (PDF no 87-0712) are observed because Ni particles can be peeled off from the Ni foam substrate as well. All the diffraction peaks of Co–NiO/Fe_2_O_3_ are indexed to these three compounds and no other crystalline phases could be detected. In contrast, clear diffraction peaks related to crystalline Co_3_O_4_ and NiO could be observed when Co–Ni based hydroxides grown on Ni foam were thermally treated under the same conditions (Fig. S5†). These results suggest that substitutional incorporation of Co into the lattices of NiO or Fe_2_O_3_ may occur during the heat treatment of the Fe–Co–Ni based precursors.

To clarify the occupancy sites of Co in the NiO or Fe_2_O_3_ lattices, the formation energies of possible substitution sites were calculated through density functional theory (DFT). Pure NiO and Fe_2_O_3_ models were constructed as shown in Fig. S6.† We then built three possible substitution sites of Co1, Co2 and Co3 on pure NiO models, and two possible substitution sites of Co1′ and Co2′ on pure Fe_2_O_3_ models, respectively (Fig. 2b–f). Obviously, the formation energy of Co substitution on NiO sites is much lower than that on Fe_2_O_3_ sites, and the formation energy is as low as 1.44 eV according to the model Co1 (Fig. 2g). This reveals that Ni in the NiO lattices is preferentially substituted by Co compared with Fe. Guided by this theoretical result, XRD Rietveld refinements were further performed, in which all the diffraction peaks coincide with the positions of the observed Bragg reflections ascribed to hexagonal Fe_2_O_3_, cubic Ni, and Co-doped cubic NiO (Fig. 2a). The low residual factors (*R*_p_ = 1.24% and *R*_wp_ = 1.58%) indicate that the XRD refinement is well-convergent, verifying the Co substitution on NiO sites. Then the corresponding structural parameters (Table S1†) can be obtained and the occupancy ratio of Co on NiO sites is about 6% in the as-prepared Co–NiO/Fe_2_O_3_ nanosheet arrays. As a contrast, the obtained XRD refinement results (Fig. S7 and Table S2†) of NiO/Fe_2_O_3_ nanosheet arrays without Co substitution indicate that partial Co substitution on NiO sites has no effect on the structure of cubic NiO.

**Fig. 3:**
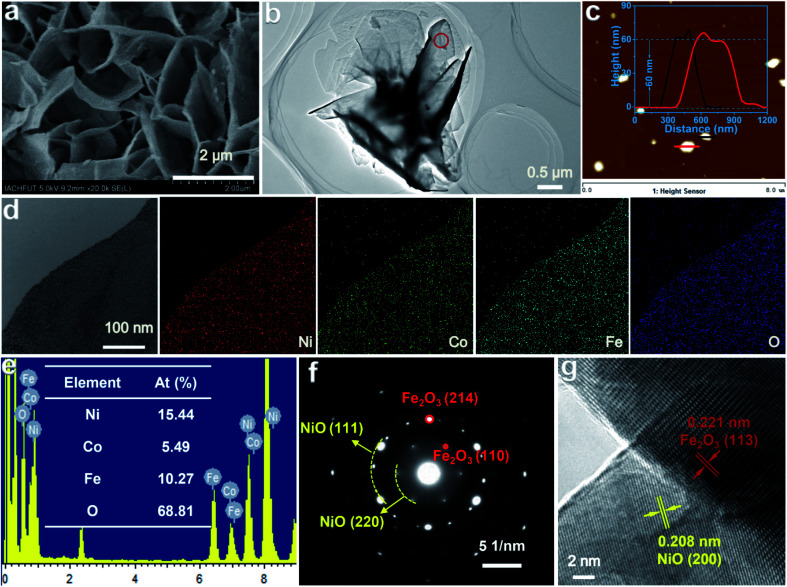
(a, b) SEM and TEM image of the Co–NiO/Fe_2_O_3_. (c) AFM image of the Co–NiO/Fe_2_O_3_ (inset: thickness profile of the Co–NiO/Fe_2_O_3_ nanosheets). (d, e) Elemental mapping result and EDS spectrum of the Co–NiO/Fe_2_O_3_. (f, g) SAED pattern and HRTEM image of the Co–NiO/Fe_2_O_3_ (red circle marked in Fig. 3b).

The morphologies and microstructures of Co–NiO/Fe_2_O_3_ were observed by electron microscopy. The as-prepared Co–NiO/Fe_2_O_3_ retains the original nanosheet morphology of the precursors (Fig. 3a and b and S8†). The atomic force microscopy (AFM) image and height profile (Fig. 3c) indicate that the Co–NiO/Fe_2_O_3_ nanosheets have an average thickness of around 60 nm. These vertically arrayed thin Co–NiO/Fe_2_O_3_ nanosheets grown on the Ni foam will allow large exposed active sites on the surface and promote the diffusion of electrolyte ions and the generated active species, thus enhancing the electrocatalytic performances of Co–NiO/Fe_2_O_3_ toward the OER. Energy dispersive spectroscopy (EDS) elemental mappings were measured to verify the elemental distributions of Co–NiO/Fe_2_O_3_. All the elements including Ni, Co, Fe, and O are uniformly distributed through the whole nanosheet (Fig. 3d). The atomic ratios of Ni, Co, Fe and O elements in the investigated catalyst are 15.44, 5.49, 10.27 and 68.81% (Ni : Co : Fe : O) according to the EDS results (Fig. 3e), which further confirm the uniform Co substitution in the Co–NiO/Fe_2_O_3_ nanosheet arrays. Similarly, NiO/Fe_2_O_3_ also presents vertically arrayed nanosheets and the elements Ni, Fe and O are distributed evenly (Fig. S9†). The selected area electron diffraction (SAED) pattern of an individual Co–NiO/Fe_2_O_3_ nanosheet shows a clear set of hexagonally arranged spots and well-defined diffraction rings (Fig. 3f), which can be assigned to Fe_2_O_3_ and NiO, respectively. Moreover, distinct lattice fringes can be identified in the high resolution transmission electron microscope (HRTEM) image, which shows that the interplanar spacing of 0.221 nm corresponds to the (113) facet of Fe_2_O_3_ and that of 0.208 nm belongs to the (200) facet of NiO, and a clear heterojunction boundary between NiO and Fe_2_O_3_ could be observed, confirming the formation of the heterostructure of NiO and Fe_2_O_3_ (Fig. 3g).

**Fig. 4:**
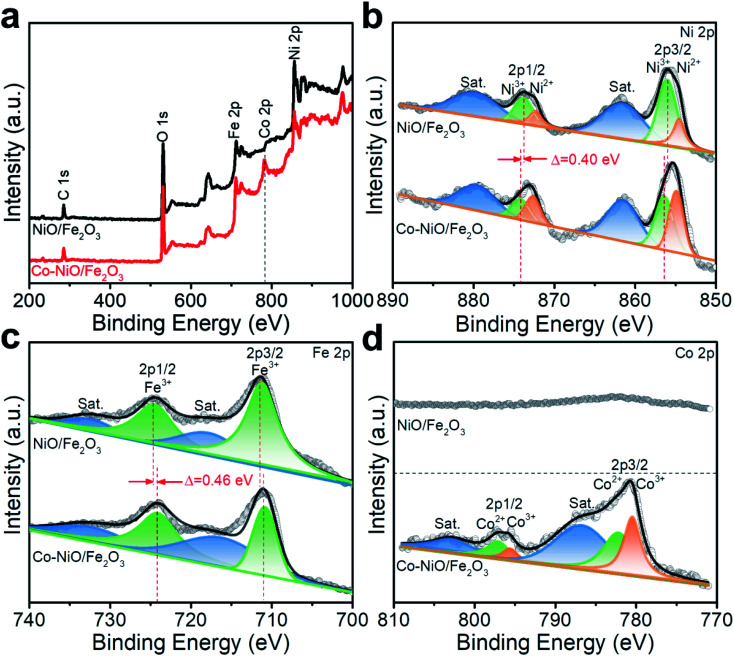
(a) Survey XPS spectra and high resolution (b) Ni 2p, (c) Fe 2p and (d) Co 2p XPS spectra of Co–NiO/Fe_2_O_3_ and NiO/Fe_2_O_3_.

Furthermore, XPS spectra were recorded to investigate the modulation of the electronic structures of the NiO/Fe_2_O_3_ nanosheet arrays *via* Co substitution. The survey XPS spectrum of the Co–NiO/Fe_2_O_3_ identifies the existence of Ni, Fe, Co, and O atoms while Co signals could not be detected in the spectrum of NiO/Fe_2_O_3_ (Fig. 4a). The high-resolution Ni 2p spectrum of the Co–NiO/Fe_2_O_3_ can be fitted into two pairs of peaks with Ni 2p_3/2_ at 854.8 eV and Ni 2p_1/2_ at 872.6 eV assigned to Ni^2+^, and Ni^3+^ is responsible for another pair of peaks with Ni 2p_3/2_ at 856.2 eV and Ni 2p_1/2_ at 874.0 eV (Fig. 4b).^36–38^ Compared with NiO/Fe_2_O_3_, both Ni 2p_1/2_ and Ni 2p_3/2_ peaks shift by 0.40 eV to higher binding energies, revealing the strong electron interaction by Co substitution.^39,40^ Fe 2p regions can be fitted into a pair of Fe^3+^ peaks at 710.8 and 724.1 eV accompanied by the satellite peaks at 717.8 and 732.2 eV in the Co–NiO/Fe_2_O_3_ (Fig. 4c).^41,42^ The spectrum of Fe 2p in Co–NiO/Fe_2_O_3_ displays a visible negative-shift of nearly 0.46 eV compared with the NiO/Fe_2_O_3_, suggesting that the electrons may transfer from Ni to Fe in the Co–NiO/Fe_2_O_3_ after introducing Co.^39,42^ For high resolution Co 2p spectra, both Co^2+^ and Co^3+^ are observed in the Co–NiO/Fe_2_O_3_. Concretely, the binding energies at 782.2 eV and 797.1 eV are assigned to Co^2+^ 2p_3/2_ and Co^2+^ 2p_1/2_, respectively (Fig. 4d), while Co^3+^ species is also observed with two characteristic peaks at 780.4 eV and 795.6 eV.^43,44^ The modulation of the electronic structures in NiO/Fe_2_O_3_ by Co substitution would provide a promising opportunity for regulating electrocatalytic OER performances, which is further discussed through DFT calculations.

### Electrocatalytic OER performance

**Fig. 5:**
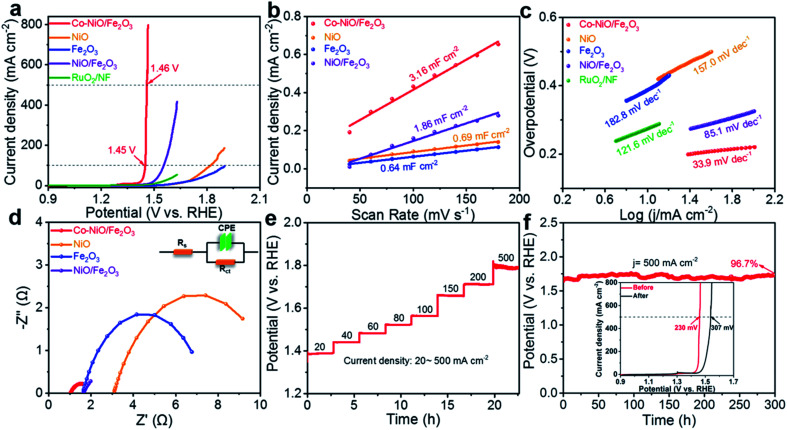
The electrochemical properties of all the electrocatalysts tested in 1 M KOH electrolyte. (a) LSV polarization curves with 90% *iR* compensation. (b) *C*_dl_ values. (c) Tafel slopes. (d) Nyquist plots of the Co–NiO/Fe_2_O_3_, NiO/Fe_2_O_3_, NiO, Fe_2_O_3_, and RuO_2_. (NiO, Fe_2_O_3_, and RuO_2_ were purchased and cast on the Ni foam for OER tests). (e) Multicurrent processes for the Co–NiO/Fe_2_O_3_. (f) Chronopotentiometry curve of the Co–NiO/Fe_2_O_3_ at a current density of 500 mA cm^−2^ (inset: LSV curves of the Co–NiO/Fe_2_O_3_ before and after 300 h chronopotentiometry).

The electrocatalytic OER activity of Co–NiO/Fe_2_O_3_ nanosheet arrays was evaluated in O_2_-saturated 1.0 M KOH electrolyte *via* a standard three-electrode configuration. In contrast, the purchased pure NiO and Fe_2_O_3_ nanoparticles were directly cast on Ni foam and used as the electrode for OER tests. As shown in Fig. 5a, an ultralow overpotential of only 220 mV at a relatively high current density of 100 mA cm^−2^ is achieved by using the Co–NiO/Fe_2_O_3_ electrocatalyst, which exhibits superior OER performance in comparison with NiO (590 mV), Fe_2_O_3_ (>669 mV), NiO/Fe_2_O_3_ (324 mV) and commercial RuO_2_ (460 mV). The as-prepared Co–NiO/Fe_2_O_3_ nanosheet arrays in this work outperform most transition metal-based electrocatalysts reported in the literature (Table S3†). More importantly, the Co–NiO/Fe_2_O_3_ also shows prompt current response with a slight augment of voltage, definitely, with a considerably low overpotential of 230 mV to deliver a high current density of 500 mA cm^−2^, which makes it possible to meet the industrial requirement. We also evaluated the OER performances of Co–NiO/Fe_2_O_3_ in alkaline simulated seawater (1 M KOH + 0.5 M NaCl) and alkaline natural seawater (1 M KOH + seawater) electrolytes. As shown in Fig. S10a,† Co–NiO/Fe_2_O_3_ retains its remarkable OER activity in different electrolytes, only requiring overpotentials of 313 mV in alkaline simulated seawater and 373 mV in alkaline natural seawater to yield a high current density of 500 mA cm^−2^, respectively. Furthermore, the electrochemical double layer capacitance (*C*_dl_) was calculated based on CV curves in the non-faradaic region to estimate the electrochemical surface area (ECSA) for different electrocatalysts (Fig. S11†). The *C*_dl_ values were calculated to be 0.69, 0.64, 1.86 and 3.16 mF cm^−2^ for NiO, Fe_2_O_3_, NiO/Fe_2_O_3_, and Co–NiO/Fe_2_O_3_, respectively (Fig. 5b), suggesting that Co–NiO/Fe_2_O_3_ can offer more abundant active sites as well as higher interfacial contact areas. The *C*_dl_ normalized linear scanning voltammetry (LSV) curves were collected to further evaluate the intrinsic catalytic activity of all the electrocatalysts (Fig. S12†). Co–NiO/Fe_2_O_3_ only requires an overpotential of 204 mV to reach 10 A F^−1^, which is significantly lower than those for the NiO, Fe_2_O_3_, and NiO/Fe_2_O_3_, demonstrating the obvious advantages of preferential Co substitution on Ni sites in the NiO/Fe_2_O_3_ nanosheet arrays. In addition, the polarization curves of seven electrodes (Fig. S13†), which were prepared in seven parallel experiments, show nearly identical overpotentials at 100, 200, and 500 mA cm^−2^, proving the superb reproducibility in our work.

The Tafel slope was observed and employed to understand the reaction kinetics of the Co–NiO/Fe_2_O_3_ during the OER process. Fig. 5c reveals a Tafel slope of 33.9 mV dec^−1^ for the Co–NiO/Fe_2_O_3_, which is significantly smaller than those of NiO (157.0 mV dec^−1^), Fe_2_O_3_ (182.8 mV dec^−1^), NiO/Fe_2_O_3_ (85.1 mV dec^−1^) and commercial RuO_2_ (121.6 mV dec^−1^), suggesting that the Co–NiO/Fe_2_O_3_ exhibits the fastest reaction kinetics. Electrochemical impedance spectroscopy (EIS) spectra were utilized to derive information on the kinetics related to the OER process. The results show that the Co–NiO/Fe_2_O_3_ (1.015 Ω) has lower charge transfer resistance (*R*_ct_) than NiO (7.818 Ω), Fe_2_O_3_ (5.834 Ω), and NiO/Fe_2_O_3_ (1.823 Ω) (Fig. 5d and Table S4†). Moreover, the Co–NiO/Fe_2_O_3_ also possesses a lower system resistance (*R*_s_) value in comparison with the NiO/Fe_2_O_3_, suggesting the higher electronic conductivity of the Co–NiO/Fe_2_O_3_ after Co substitution. Partial density of states (DOS) was further studied to understand the improvement of electronic conductivity.^45,46^ Fig. S14† shows partial DOS plots before and after Co substitution. Asymmetric DOS is observed due to magnetic Ni atoms. A semiconductor nature, where the band gap of the electrons is wide and has a semiconducting band structure, is noted for pure NiO. After Co substitution on Ni sites in NiO, a significant narrower band gap is observed (from 0.80 eV to 0.42 eV). This reveals that the electronic conductivity of the NiO/Fe_2_O_3_ can be substantially enhanced with the introduction of Co atoms into NiO. Accordingly, the fast reaction kinetics of the Co–NiO/Fe_2_O_3_ can be attributed to the short ion diffusion pathway resulting from vertically arrayed nanosheets and fast electron transport enabled by Co-doped NiO/Fe_2_O_3_.

Long-term stability is also an important aspect to evaluate electrocatalysts. The voltages of the Co–NiO/Fe_2_O_3_ electrode can be stabilized quickly and maintain a stable value for 10 000 seconds at different applied current densities (from 20 mA cm^−2^ to 500 mA cm^−2^) (Fig. 5e). Such a response implies the high electrocatalytic stability of the Co–NiO/Fe_2_O_3_ electrode. Moreover, the Co–NiO/Fe_2_O_3_ almost maintains a constant potential value (∼1.72 V *vs.* RHE) for 300 h at a high current density of 500 mA cm^−2^ with a retention rate of 96.7% (Fig. 5f) and the inset of Fig. 5f exhibits the overpotential without obvious change after 300 h chronopotentiometry. The real-time potential of the Co–NiO/Fe_2_O_3_ is also highly stable at such a high current density of 500 mA cm^−2^ throughout 50 h of continuous operation in either an alkaline simulated seawater or alkaline natural seawater electrolyte, further demonstrating its remarkable durability at large current densities for practical application (Fig. S10b†). To further demonstrate the stability, we examined the morphology of the Co–NiO/Fe_2_O_3_ after the OER test for long hours. The Co–NiO/Fe_2_O_3_ retains its original nanosheet structure (Fig. S15a and c†), revealing the strong adhesion between the nanosheets and the substrate as well as the reliable mechanical stability of the electrode. Furthermore, XPS spectra verify the presence of Ni, Co, and Fe after the OER test, where the relative intensities of peaks belonging to Ni^3+^ and Co^3+^ are significantly increased (Fig. S16†), indicating that Ni and Co are readily oxidized to higher valence states. These results demonstrate that the as-prepared Co–NiO/Fe_2_O_3_ in our work could act as a robust OER electrocatalyst with low overpotential and long-term stability at a high current density (>500 mA cm^−2^), which shows its great potential for water splitting anode materials in practical use (Table S3†).

We established a hybrid overall water splitting system for hydrogen production in 1.0 M KOH media, in which the prepared Co–NiO/Fe_2_O_3_ electrode and the commercial Pt/C catalyst were used as the anode and cathode, respectively. The LSV polarization curve reveals that only a low voltage of 1.64 V is required to reach a current density of 500 mA cm^−2^ for this system, while the water splitting cell consisting of commercial RuO_2_ and Pt/C catalysts needs a voltage of 1.65 V at 10 mA cm^−2^ (Fig. S17a†). Furthermore, the above system (Co–NiO/Fe_2_O_3_‖Pt/C) has good stability without visible changes in the operating voltage after electrolysis of water at 500 mA cm^−2^ for at least 50 h (Fig. S17b†).

### Density functional theory calculations

**Fig. 6:**
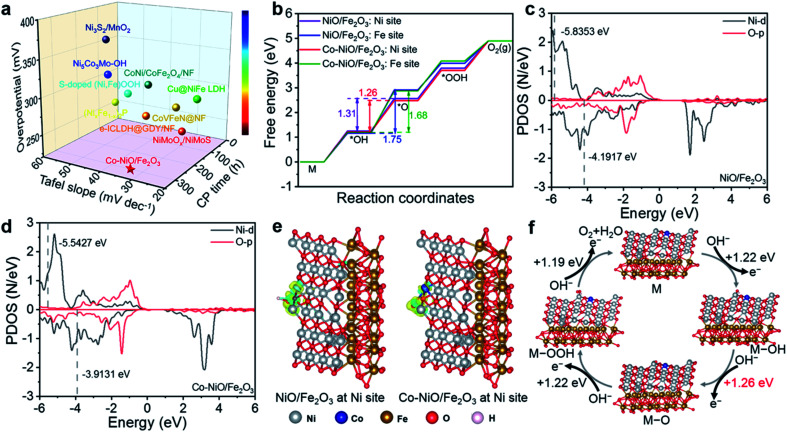
(a) OER performances of the Co–NiO/Fe_2_O_3_ in comparison with other advanced transition metal-based electrocatalysts in 1.0 M KOH alkaline solution. (Overpotentials and chronopotentiometry time were obtained at 100 mA cm^−2^ and 500 mA cm^−2^, respectively.) (b) Free energy diagram for the OER at Ni and Fe sites on the surface model of the NiO/Fe_2_O_3_ and Co–NiO/Fe_2_O_3_. PDOS of Ni d orbitals for the (c) NiO/Fe_2_O_3_ and (d) Co–NiO/Fe_2_O_3_ at *OH intermediates. (e) The side views of charge density differences for *OH adsorbed on the Ni in the NiO/Fe_2_O_3_ and Co–NiO/Fe_2_O_3_. (The isosurface is 0.05 e Å^−3^. Yellow and cyan regions represent charge accumulation and depletion, respectively.) (f) OER cycle for the Co–NiO/Fe_2_O_3_ on the Ni sites.

In general, the Co–NiO/Fe_2_O_3_ nanosheet arrays as an OER electrocatalyst not only exhibit admirable OER performance in terms of overpotential and Tafel slope, but also show an extra-long stability at large current densities, which outperforms many other advanced transition metal-based electrocatalysts, as illustrated in Fig. 6a. To get an insight into the superb OER activity, DFT calculations have been carried out to unravel the structure–activity correlation of Co–NiO/Fe_2_O_3_ with Co substitution during the OER process. As shown in Fig. 6b, the Gibbs free energies (Δ*G*) of four OER elementary reactions (M → *OH → *O → *OOH → O_2_) at Ni and Fe sites in the NiO/Fe_2_O_3_ and Co–NiO/Fe_2_O_3_ with Co substitution were calculated. The elementary reaction with the maximum change of Δ*G* is identified as the rate-determining step (RDS) in the OER process,^47^ so the second step from *OH to *O is considered as the RDS for both NiO/Fe_2_O_3_ and Co–NiO/Fe_2_O_3_ in our work (Fig. S18†). It is noteworthy that Co substitution in the Co–NiO/Fe_2_O_3_ significantly reduces the energy barriers of the RDS for both Ni (from 1.31 eV to 1.26 eV) and Fe (from 1.74 eV to 1.68 eV) sites, thus showing the best theoretical OER activity (Fig. 6b). Furthermore, the energy barrier of RDS at Ni sites in the Co–NiO/Fe_2_O_3_ is significantly lower than that at Fe sites. That is to say that the RDS intermediate may form toxic adsorption at the Fe sites. This result implies that the Ni sites become main active sites in the OER process.^48^ Meanwhile, the projected density of states (PDOS) of Ni d orbitals and Fe d orbitals on the *OH intermediate were also calculated before and after Co substitution in NiO/Fe_2_O_3_, respectively.^49^ The d-band center of Ni is −5.5427 eV and that of Fe is −7.1802 eV with respect to the Fermi energy level in the Co–NiO/Fe_2_O_3_. In the case of NiO/Fe_2_O_3_, the d-band center of Ni is −5.8353 eV and that of Fe is −7.3221 eV relative to the Fermi energy level (Fig. 6c and d and S19†). In other words, the d-band centers of Ni and Fe in the Co–NiO/Fe_2_O_3_ are biased toward the Fermi energy level, while those in the NiO/Fe_2_O_3_ are deviated from the Fermi energy level, revealing the better OER activity after Co substitution on Ni sites. Besides, the closer d-band center of Ni in the Co–NiO/Fe_2_O_3_ relative to the Fermi energy level further confirms that the Ni sites in the Co–NiO/Fe_2_O_3_ are the main OER active sites.

Since the second step from *OH to *O has been determined as the RDS in our work, we further investigated the charge density differences for *OH adsorbed models at the Ni sites in the NiO/Fe_2_O_3_ and Co–NiO/Fe_2_O_3_. The Co–NiO/Fe_2_O_3_ has more accumulated charge at the interface of the *OH substrate than the NiO/Fe_2_O_3_, demonstrating the stronger adsorption of *OH for the Co–NiO/Fe_2_O_3_ with Co substitution (Fig. 6e). Moreover, as shown in Fig. 6e, more electrons transferred from the O–H *σ*_sp_ bonding orbital after Co substitution on Ni sites in the Co–NiO/Fe_2_O_3_ accelerate the dissociation of the O–H bond and the formation of *O species, which is beneficial for the lower energy barrier of the RDS.^50,51^ Based on these results, we propose the catalytic mechanism of the OER on the Co–NiO/Fe_2_O_3_ electrocatalyst (Fig. 6f). The Co substitution on Ni sites causes the regulation of the d-band center of the Co–NiO/Fe_2_O_3_, which results in a significant decrease in the energy barrier of the RDS (from *OH to *O) in the OER process, further enhancing the OER activity.

## Conclusion

In summary, NiO/Fe_2_O_3_ nanosheet arrays with preferential Co substitution on Ni sites have been successfully prepared by a hydrothermal reaction and subsequent heat treatment. DFT calculations prove that the incorporation of Co into Ni sites of the NiO/Fe_2_O_3_ possesses the lowest formation energies among possible substitution sites, and XRD Rietveld refinements reveal that about 6% of Ni is substituted by Co in the NiO/Fe_2_O_3_. The Co substitution in the NiO/Fe_2_O_3_ can effectively tune the electronic structures, leading to the improvement of the electronic conductivity and d-band center energy level, and the reduction of the energy barriers for the rate-determining step in the OER process as confirmed by DFT calculations. The vertically aligned nanosheet structure also allows a short ion diffusion length in the OER process. As a result, the Co–NiO/Fe_2_O_3_ nanosheet arrays as an electrocatalyst exhibit high activity, fast reaction kinetics, and superb stability toward the OER. In detail, only a low overpotential of 230 mV is required to achieve a high current density of 500 mA cm^−2^, and superior stability with negligible activity decay could be realized at such a high current density of 500 mA cm^−2^ for 300 h continuous operation. The Tafel slope is also as small as 33.9 mV dec^−1^. This finding in our work manifests the effectiveness of preferential Co substitution in the Ni–Fe based oxides to tune the 3d electrons and improve the electrocatalytic activity to meet the industrial demands. This work also provides an effective strategy to guide the rational design of high-activity and robust transition metal oxide based OER electrocatalysts *via* morphology and electronic structure modulation.

## Data availability

All relevant data are presented in the paper and the ESI.†

## Author contributions

Yuping Lin conducted the synthesis, structural analysis and OER electrocatalytic performance measurements, and wrote the draft. Mengqiu Huang assisted with the synthesis and data analysis. Xiaoming Fan, Zeheng Yang, and Weixing Zhang supervised the work and revised the manuscript. All authors participated in the discussion of the results.

## Conflicts of interest

The authors declare no competing financial interest.

## Supplementary Material

SC-013-D2SC02019J-s001
