# Supplementary material for: Preferential Co substitution on Ni sites in Ni–Fe oxide arrays enabling large-current-density alkaline oxygen evolution
Source: Chem Sci. 2022 May 31;13(24):7332–40. doi: 10.1039/d2sc02019j (PMC9214842; doi:10.1039/d2sc02019j)
Supplement: SC-013-D2SC02019J-s001 [file SC-013-D2SC02019J-s001.pdf]

Electronic Supplementary Material (ESI) for Chemical Science.
This journal is © The Royal Society of Chemistry 2022

Supporting Information

Preferential Co substitution on Ni sites in Ni-Fe oxide arrays enabling large-current-density alkaline oxygen evolution

Yuping Lin, Xiaoming Fan,* Mengqiu Huang, Zeheng Yang, Weixin Zhang*

School of Chemistry and Chemical Engineering, Anhui Key Laboratory of Controllable Chemical Reaction and Material Chemical Engineering, Hefei University of Technology Hefei 230009, PR China

Institute of Energy, Hefei Comprehensive National Science Center, Anhui, Hefei 230009, PR China

*Corresponding authors: xmfan@hfut.edu.cn, wxzhang@hfut.edu.cn

Experimental Section

Chemicals

$Ni(NO_3)_2 \bullet 6H_2O$ (⩾99.0%, AR), $Co(NO_3)_2 \bullet 6H_2O$ (⩾99.0%, AR), $FeSO_4 \bullet 7H_2O$ (⩾99.0%, AR), NaCl (⩾99.0%, AR), RuO_2 (99.9%), H_2SO_4 and acetone were purchased from Sinopharm Chemical Reagent Co., Ltd. Nafion solution (5 wt %) was purchased from SigmaAldrich Chemical Reagent Co., Ltd. Ni foam was purchase from Shenzhen Meisen electromechanical equipment Co., Ltd. All of the reagents were used without further purification.

Materials Synthesis

Synthesis of Co-NiO/Fe_2O_3 Nanosheet Arrays

In this experiment, Ni foam with geometric area of 2×2 cm^2 was firstly washed by acetone, ethanol, and H_2SO_4 aqueous solution (2.0 M), respectively. For the synthesis of Co-NiO/Fe_2O_3 catalyst, the pre-cleaned Ni foam and the mixed solution (20 mL) containing $Co(NO_3)_2 \bullet 6H_2O$ (12.5 mM) and $FeSO_4 \bullet 7H_2O$ (12.5 mM) were successively transferred to a 50 mL Teflon-lined autoclave and kept at 80 °C for 12 hrs. Then, the sample was washed with deionized water and absolute ethanol for several times to get hydroxide nanosheet precursors after drying overnight at 60 °C. Subsequently, the Co-NiO/Fe_2O_3 was obtained by calcination the above precursor in air at 450 °C for 2 hrs. The mass loading of the catalysts supported on Ni foam substrate are around 2.15 mg cm^{-2}.

Synthesis of NiO/Fe_2O_3 Nanosheet Arrays

The NiO/Fe_2O_3 nanosheet arrays were prepared by the same procedure for the Co-NiO/Fe_2O_3 nanosheet arrays except that $Ni(NO_3)_2 \bullet 6H_2O$ (12.5 mM) to replace $Co(NO_3)_2 \bullet 6H_2O$ (12.5 mM).

Materials Characterizations

The composition and structure of the as-prepared samples were examined by X-ray diffraction (XRD) on a X-ray diffractometer (SmartLab, Rigaku, Japan) using a Cu Kα radiation source (λ= 0.154178 nm). Field-emission scanning electron microscope (FESEM) (SU8020, Hitachi, Japan) and transmission electron microscope (TEM) (JEM-1400flash, JEOL, Japan) images of the

samples were obtained to characterize the morphology and microstructure. The thickness of the sample was measured by atomic force microscopy (AFM) (Dimension Icon, Bruker, Germany). Elemental mapping and select area electron diffraction (SAED) patterns of the samples were taken on a field-emission transmission electron microscope (FETEM) (JEM-2100F, JEOL, Japan). The valence state of the samples was recorded by X-ray photoelectron spectroscopy (XPS) (ESCALAB250Xi, Thermo, America) equipped with a hemispherical energy analyzer and a monochromatic Al Kα source.

Electrochemical Measurements

All electrochemical measurements were carried out on an electrochemical workstation (CHI 760E) via a conventional three-electrode system in 1.0 M O_2-saturated KOH media under ambient conditions. 1 M KOH + 0.5 M NaCl and 1 M KOH + seawater were used to evaluate the OER performance for actual industrial application and the natural seawater was collected from Bohai Sea, China. A Pt net (1×1.5 cm^2), an Hg/HgO electrode, and the as-prepared catalysts were employed as the counter, reference electrode and working electrode, respectively. The linear sweep voltammetry (LSV) curves were collected at a scan rate of 5 mV s^{-1} with 90% iR compensation. The C_{dl} values were evaluated based on cyclic voltammetry (CV) curves under non-Faradaic region. Electrochemical impedance spectroscopy (EIS) was investigated in an O_2-saturated electrolyte with amplitude of 5 mV and the frequency range from 10 kHz to 0.01 Hz. The multicurrent processes and chronopotentiometry curve were investigated without iR compensation.

For comparison, the RuO_2, NiO, Fe_2O_3 were evaluated. The working electrodes of these catalysts were prepared by dispersing 2 mg powders into a water–ethanol solution containing 10 μL Nafion, respectively, and sonicating for 1 h to form a homogeneous catalyst inks. Then the catalyst ink was cast onto Ni foam and dried in air at room temperature.

Density Functional Theory Calculations

Quantum espresso (QE) based on the pseudopotential plane wave (PPW) method was used to complete density functional theory (DFT) calculations.[1,2] The perdew-Bueke-Ernzerhof (PBE) functional was employed to describe exchange-correlation effects of electrons.[3] Projected augmented wave (PAW) potentials were chosed to describe the ionic cores and take valence

electrons into account using a plane wave basis set with a kinetic energy cutoff of 500 eV.[4,5] Slab models were built by slicing (111) and (110) planes of NiO and Fe_2O_3 respectively to model the surfaces of materials. An extra vacuum of 12 A were applied along z-direction to avoid interactions. In order to specify the most stable doping sites for Co, different possible sites were calculated in NiO and Fe_2O_3, where the most stable ones were used to carry out further simulation. Further, to explore the effects of NiO/Fe_2O_3, heterojunction models were built considering both un-doped and Co-doped structures. All of the structures were first optimized to reach their most stable configuration. All of the atom positions were allowed to relax during the geometry optimizations. The Brillouin-zone sampling was conducted using Monkhorst-Pack (MP) grids of special points with the separation of 0.04 $Å^{-1}$.[6] The convergence criterion for the electronic self-consistent field (SCF) loop was set to 1×10^{-5} eV/atom. The atomic structures were optimized until the residual forces were below 0.05 eV $Å^{-1}$. Based on the relaxed structures, free energy along OER reaction path was calculated. The d-band center was calculated based on the following equation:

$$\epsilon = \frac{\int_{-\infty}^{E_f} E f_d(E) dE}{\int_{-\infty}^{E_f} f_d(E) dE}$$

Where f_d represent the density of states (DOS) of the specified orbital.

Differential charge density was calculated by $D_{diff} = D_{tot} - D_{sub} - D_{abs}$, where D_{tot}, D_{sub} and D_{abs} were charge density of whole system, density of substrate and density of absorbed molecules respectively.

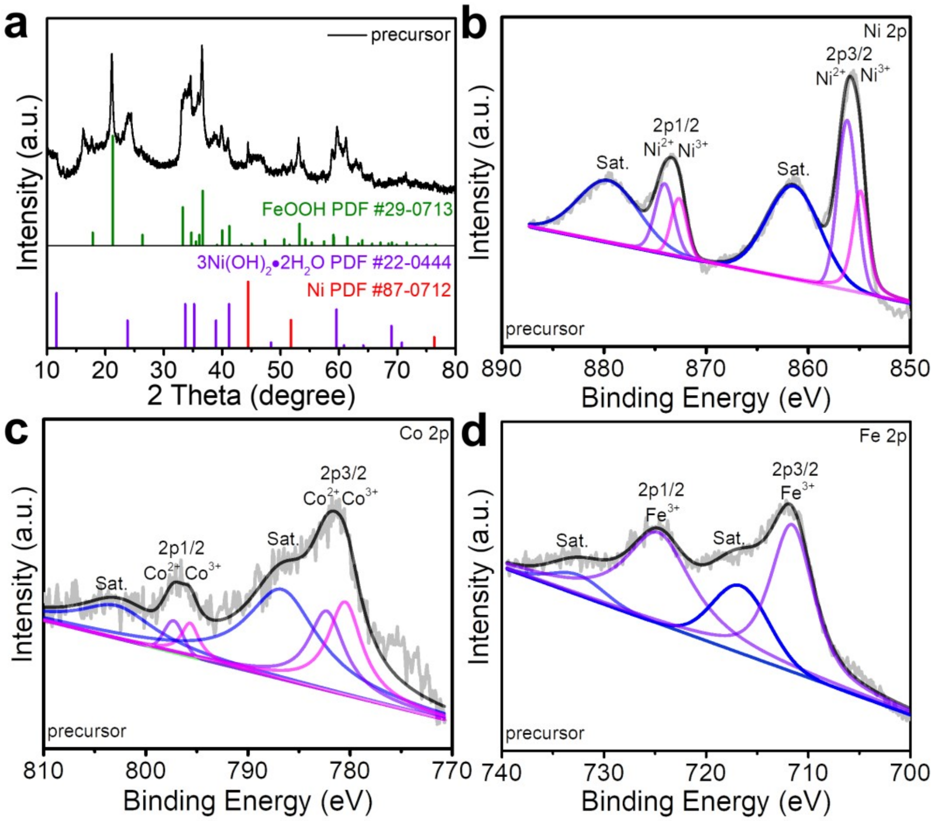

Fig. S1 (a) XRD pattern and XPS spectra of (b) Ni 2p, (c) Co 2p and (d) Fe 2p for the obtained precursor after hydrothermal reaction of Ni foam, $Co(NO_3)_2$ and $FeSO_4$.

Fig. S1a reveals the formation of $3Ni(OH)_2 \bullet 2H_2O$ and FeOOH precursors after hydrothermal reaction containing both Co^{2+} and Fe^{2+}. The formation of FeOOH may be due to that the hydrolysis product $Fe(OH)_2$ could be easily oxidized to FeOOH by the dissolved oxygen. Furthermore, the Co signals could be detected in the XPS spectra although the corresponding diffraction peaks related to Co could not observed in the XRD patterns for the precursors. The high-resolution Co 2p and Ni 2p spectra of the precursors can be assigned to Ni^{2+}, Ni^{3+} and Co^{2+}, Co^{3+}, respectively, and Fe 2p regions can be fitted into a pair of Fe^{3+} peaks and satellite peaks in the precursors (Fig. S1b-d).

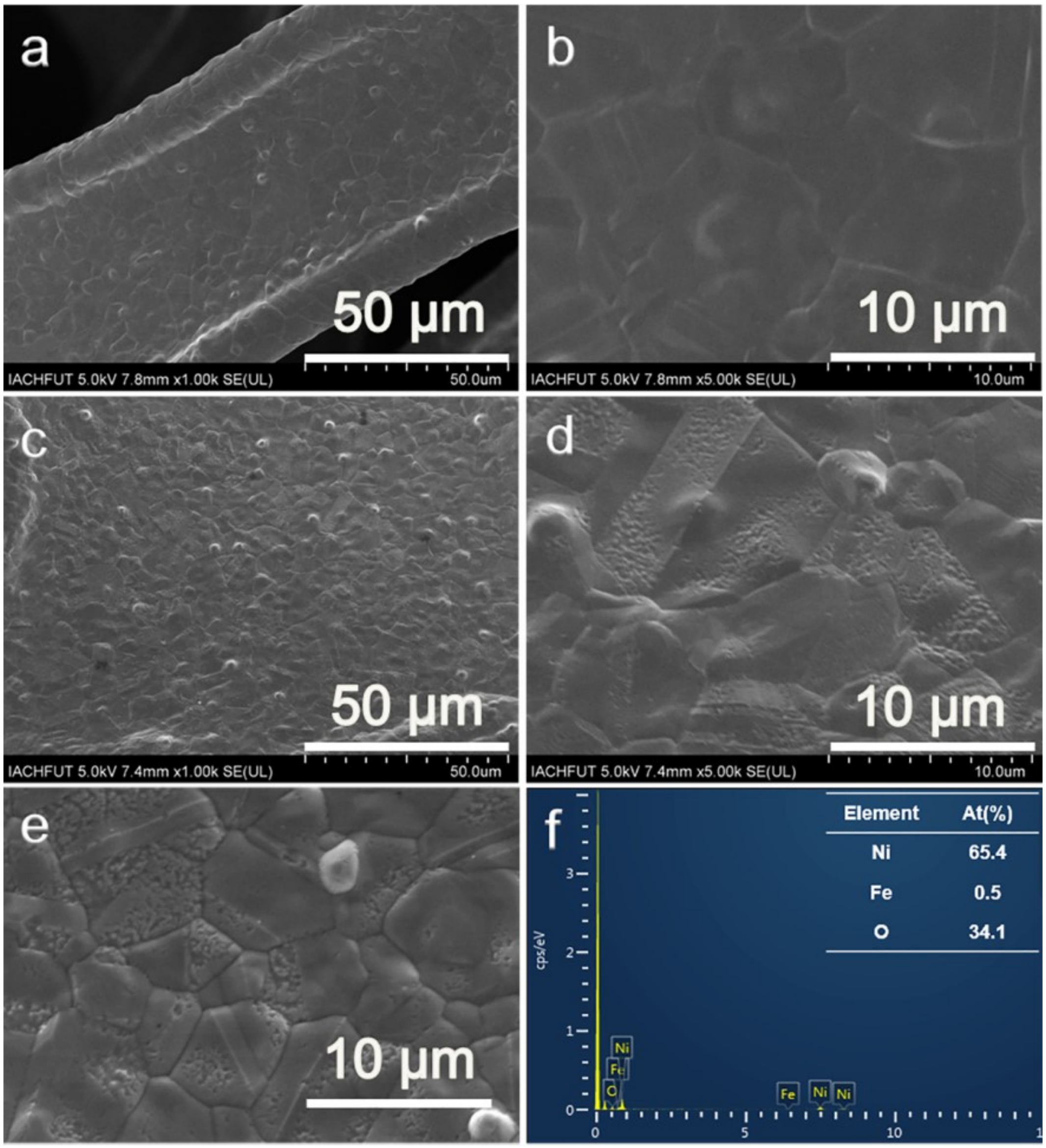

Fig. S2 (a-b) FESEM images of pure Ni foam. (c-d) FESEM images, (e) SEM and (f) the corresponding EDS spectrum of the obtained Ni foam after hydrothermal reaction of Ni foam and $FeSO_4$ aqueous solution (without the addition of $Co(NO_3)_2$).

When only Ni foam and $FeSO_4$ are added into the hydrothermal system, as shown in the Fig. S2, the FESEM images exhibit that no nanosheets or other nanostructures are observed on the surface of the skeleton of smooth Ni foam, which is also confirmed by EDS results, suggesting that the co-deposition reaction cannot occur without Co^{2+} ions assistance.

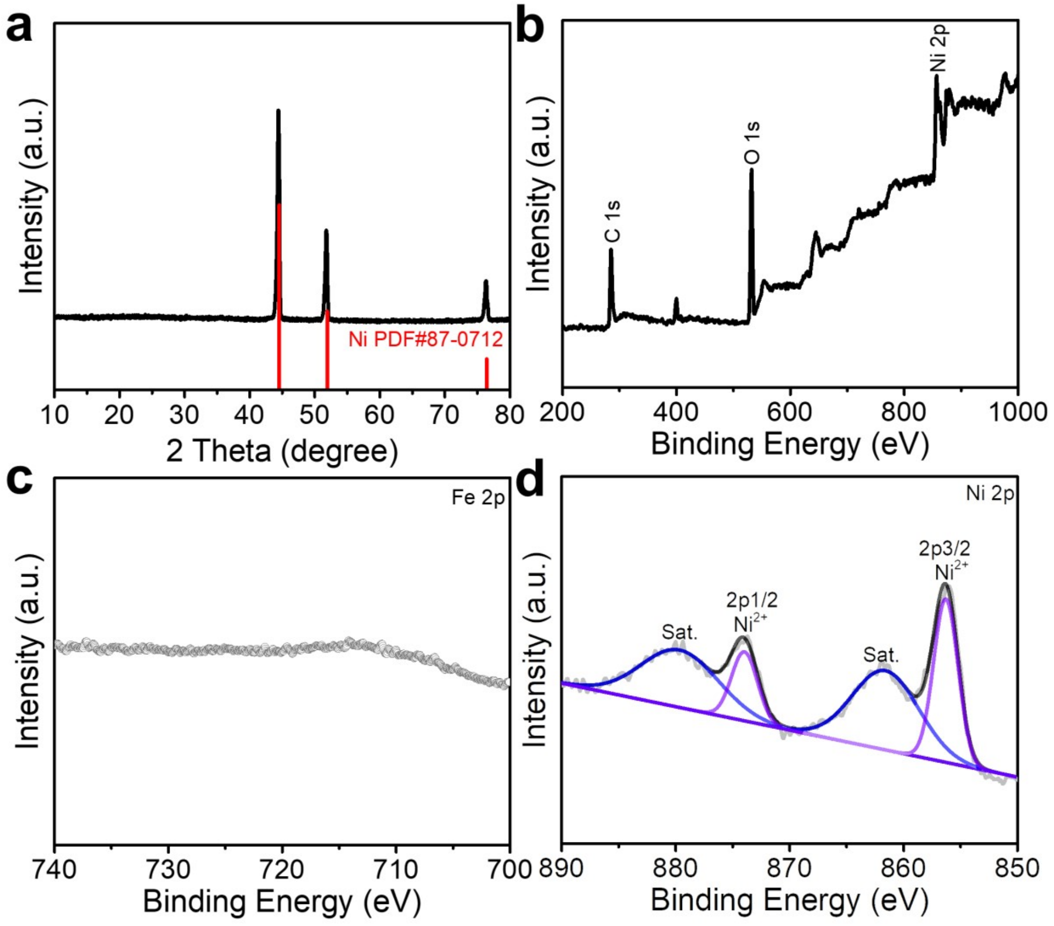

Fig. S3 (a) XRD pattern, (b) survey XPS spectrum and high resolution (c) Fe 2p and (d) Ni 2p XPS spectra for the obtained Ni foam after hydrothermal reaction of Ni foam and $FeSO_4$ aqueous solution (without the addition of $Co(NO_3)_2$).

In the absence of Co^{2+}, only the diffraction peaks of Ni foam substrate could be observed after hydrothermal reaction (Fig. S3a), and no nanosheet arrays or other nanostructures could be observed on Ni foam (Fig. S2). Besides, the survey and Fe/Ni 2p XPS spectra confirm the existence of Ni and O elements while Fe signals could not be detected (Fig. S3b-d).

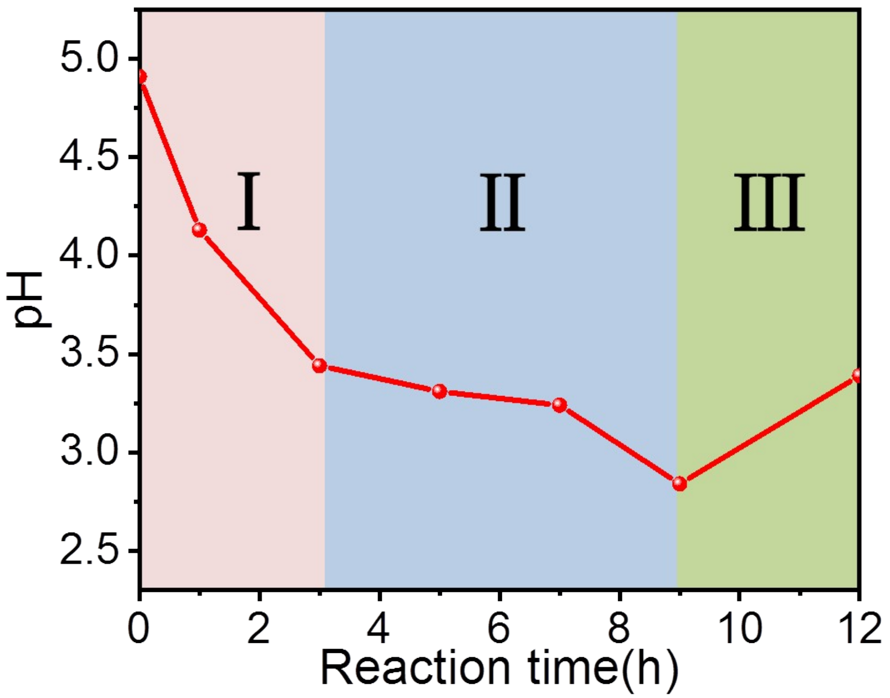

Fig. S4 The pH values of the solution for the synthesis of hydroxide precursors in hydrothermal reaction.

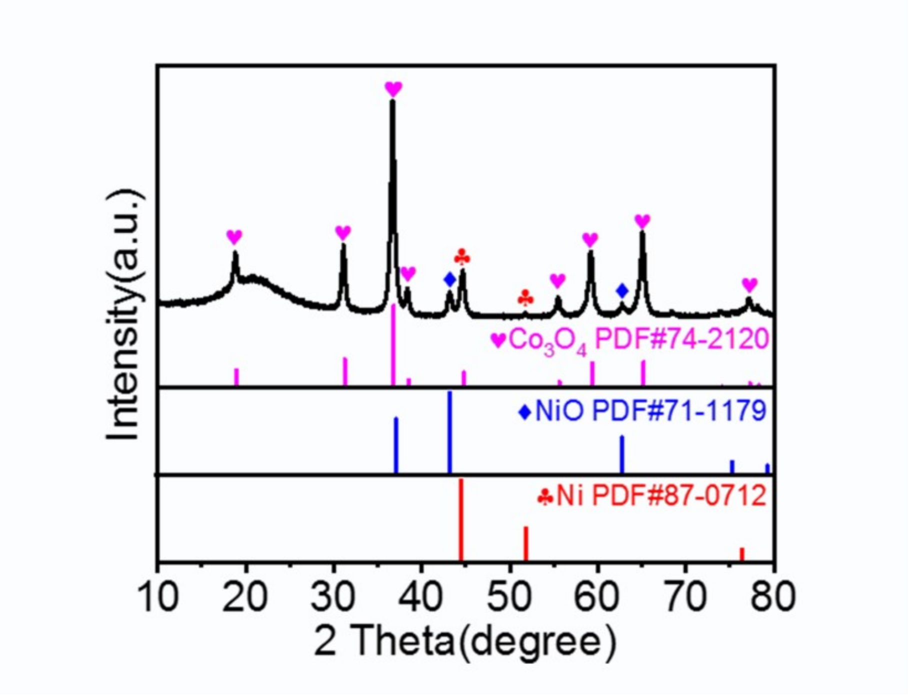

Fig. S5 XRD pattern of NiO/Co_3O_4.

$Co(NO_3)_2$•$6H_2O$ and Ni foam were used to prepare Co-Ni based hydroxides under hydrothermal condition. Then, the hydroxide precursors were further treated in air at 450 °C for 2 hrs. Crystalline Co_3O_4 and NiO phases could be detected in the XRD pattern. In contrast, no crystalline Co_3O_4 could be found in the Co-NiO/Fe_2O_3. This suggests that substitutional incorporation of Co into the lattices of NiO or Fe_2O_3 may occur during the heat treatment of Fe-Co-Ni based hydroxide precursors.

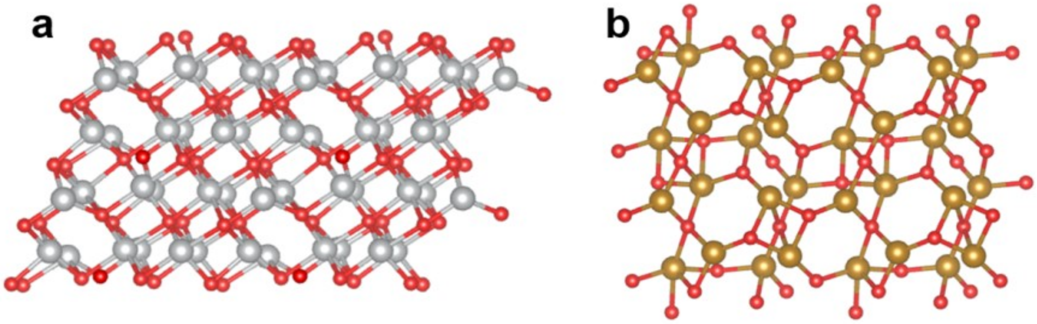

Fig. S6 The top views of (a) pure NiO and (b) pure Fe_2O_3.

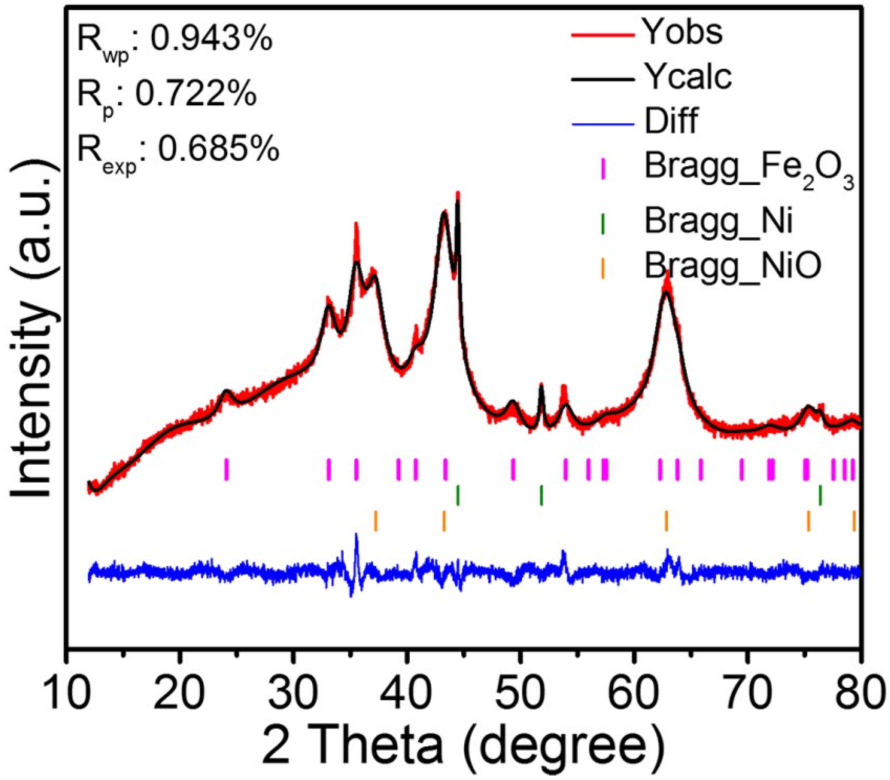

Fig. S7 Rietveld refined XRD pattern of NiO/Fe_2O_3.

The XRD pattern of NiO/Fe_2O_3 is refined, which confirms that the as-prepared NiO/Fe_2O_3 also consists of NiO, Fe_2O_3, and Ni compared with the Co-NiO/Fe_2O_3 (Ni was peeled from Ni foam.)

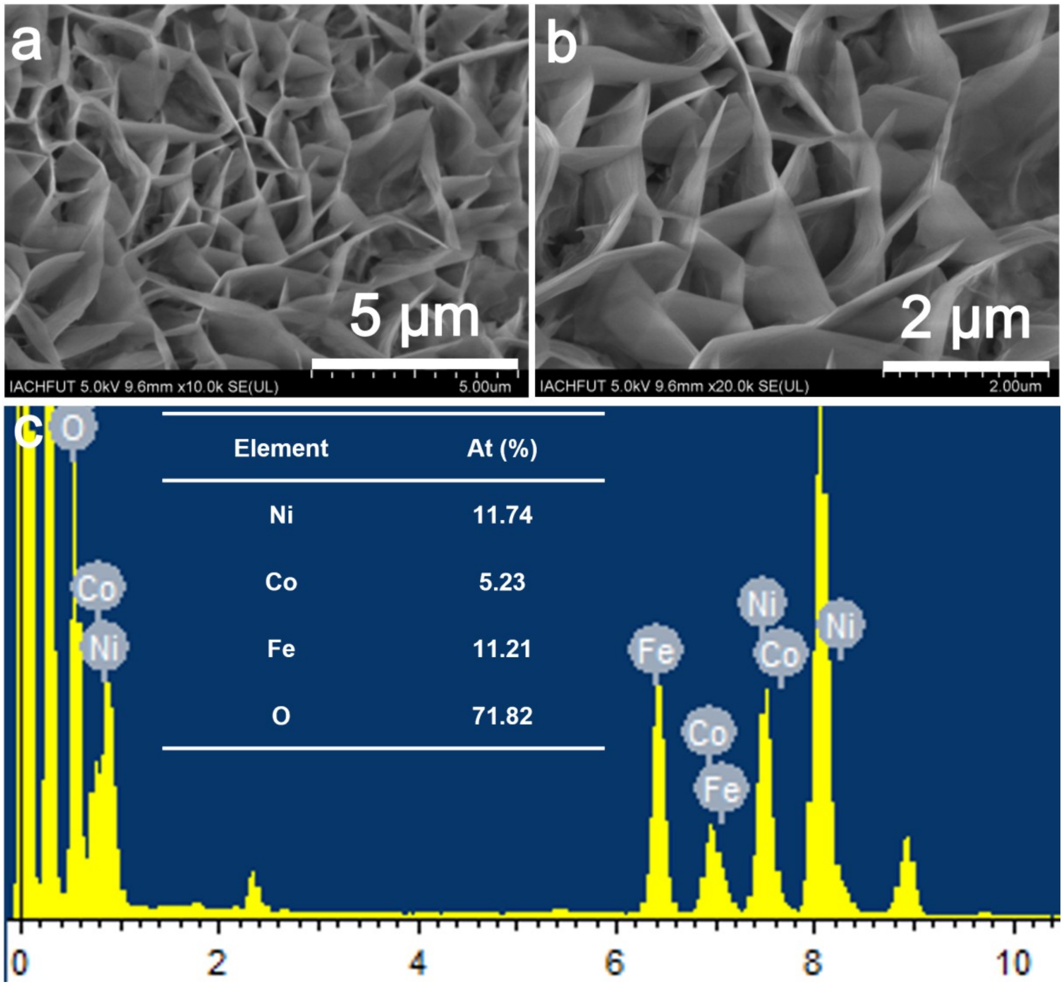

Element	At (%)
Ni	11.74
Co	5.23
Fe	11.21
O	71.82

Fig. S8 (a, b) FESEM images and (c) EDS spectrum of the obtained precursor after hydrothermal reaction of Ni foam, $Co(NO_3)_2$ and $FeSO_4$.

The Fe-Co-Ni based precursors exhibit typical vertically arrayed nanosheet morphology with Ni, Co, Fe and O elements.

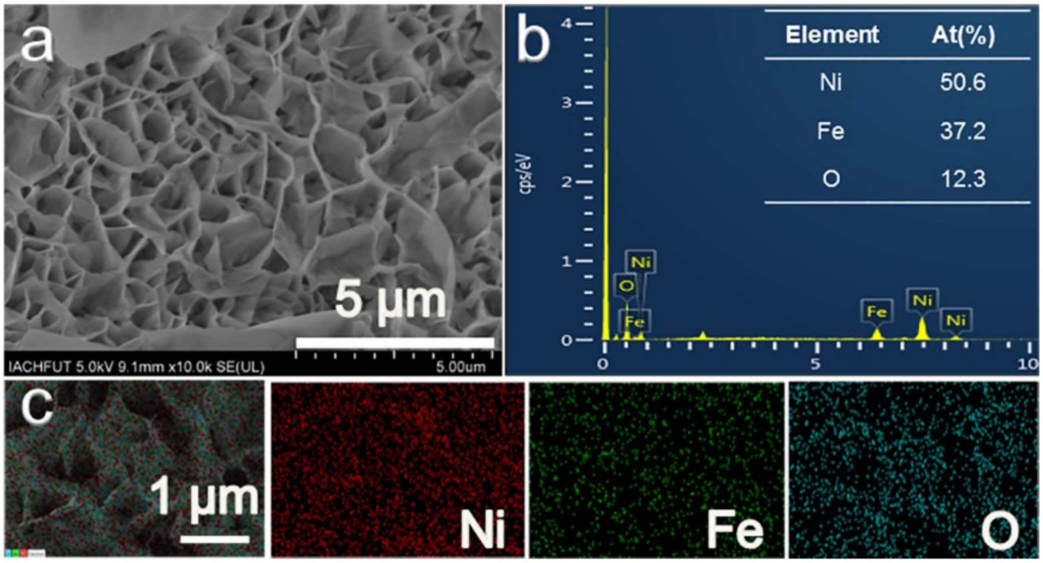

Fig. S9 (a) FESEM image, (b) EDS spectrum, and (c) elemental mapping images of the NiO/Fe_2O_3.

We used the similar method to prepare the Ni and Fe based precursors with the addition of Ni^{2+} instead of Co^{2+} in the hydrothermal reaction. The obtained precursors were then calcined at the same condition. The resulting NiO/Fe_2O_3 also maintains the morphology of nanosheets and shows uniform elemental distribution.

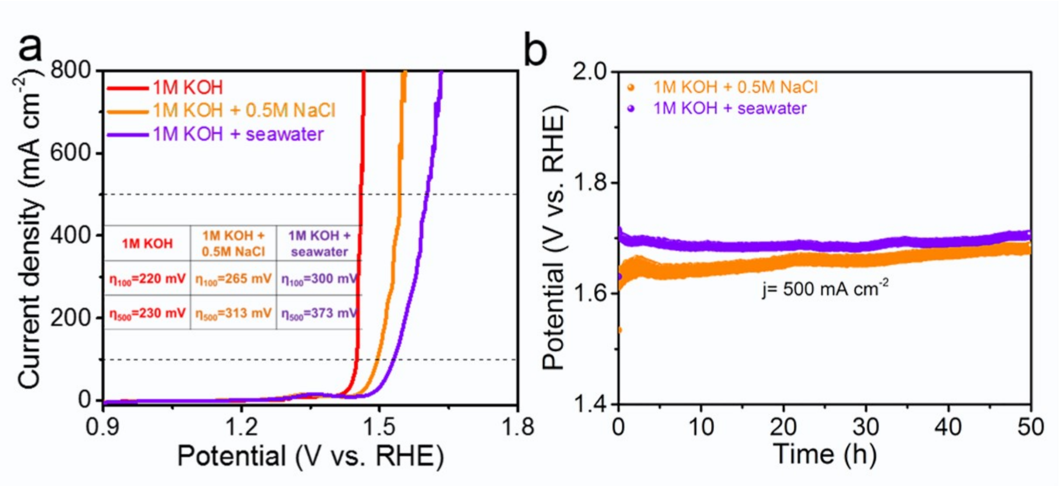

Fig. S10 (a) Polarization curves and (b) long-term stability tests at a constant current density of 500 mA cm^{-2} for the Co-NiO/Fe_2O_3 electrode in different electrolytes.

Alkaline simulated seawater (1 M KOH + 0.5 M NaCl) and alkaline natural seawater (1 M KOH + seawater) electrolytes were used to further evaluate the OER performance of the Co-NiO/Fe_2O_3. The Co-NiO/Fe_2O_3 shows overpotentials of 265 and 300 mV at 100 mA cm^{-2} in alkaline simulated seawater, and 313 and 373 mV at 500 mA cm^{-2} in alkaline natural seawater. The Co-NiO/Fe_2O_3 also maintains stable potentials at 500 mA cm^{-2} under continuous operation for 50 hrs both in either alkaline simulated seawater or alkaline natural seawater.

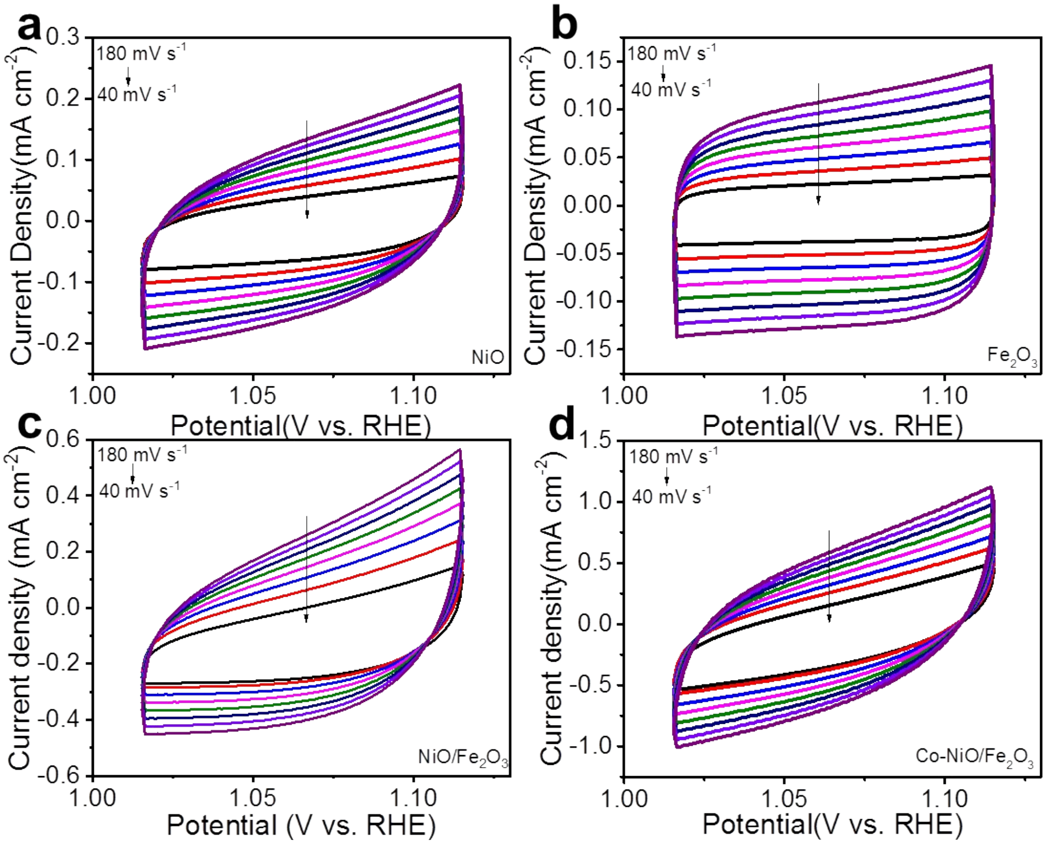

Fig. S11 CV curves of different catalysts under various scan rates (40 to 180 mV s^{-1}). (a) NiO, (b) Fe_2O_3, (c) NiO/Fe_2O_3, (d) Co-NiO/Fe_2O_3.

CV curves of different catalysts NiO, Fe_2O_3, NiO/Fe_2O_3 and Co-NiO/Fe_2O_3 have been added as shown in Fig. S11. We calculated the C_{dl} values of different electrocatalysts based on these CV curves and further estimated the electrochemical surface areas for different electrocatalysts. The C_{dl} values were calculated to be 0.69, 0.64, 1.86 and 3.16 mF cm^{-2} for NiO, Fe_2O_3, NiO/Fe_2O_3, and Co-NiO/Fe_2O_3, respectively, suggesting that the Co-NiO/Fe_2O_3 can offer more abundant active sites as well as higher interfacial contact areas.

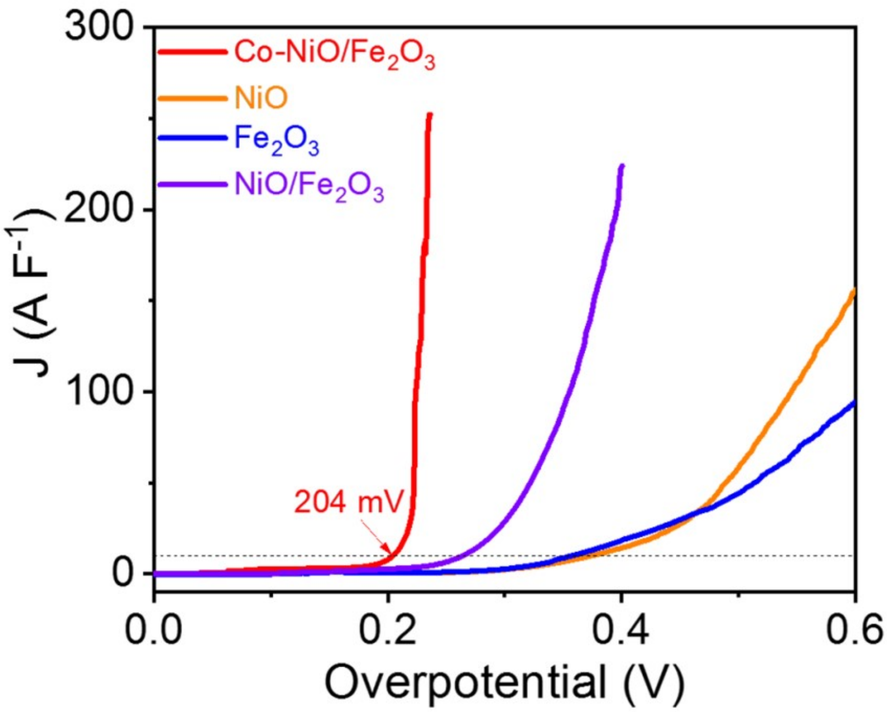

Fig. S12 Linear sweep voltammetry curves normalized by electrochemical double-layer capacitance.

To further evaluate the intrinsic catalytic activity of all the catalysts, the electrochemical double layer capacitance (C_{dl}) normalized linear scanning voltammetry (LSV) curves were collected. The Co-NiO/Fe_2O_3 requires only 204 mV overpotential to reach 10 A F^{-1}, which is significantly lower than those for the NiO, Fe_2O_3 and NiO/Fe_2O_3, demonstrating the high intrinsic OER activity of the Co-NiO/Fe_2O_3 with Co substitution.

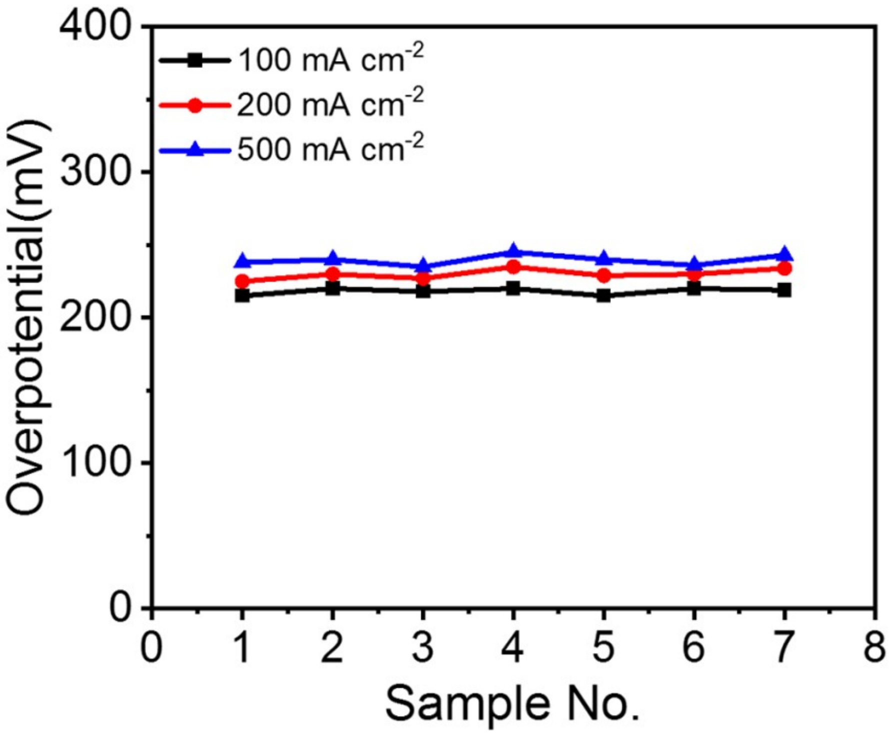

Fig. S13 Overpotentials at current densities of 100, 200, and 500 mA cm^{-2} for seven Co-NiO/Fe_2O_3 electrodes prepared in seven parallel experiments.

Seven Co-NiO/Fe_2O_3 electrodes exhibit nearly identical potentials at 100, 200 and 500 mA cm^{-2} in seven parallel experiments, demonstrating the superb reproducibility in this work.

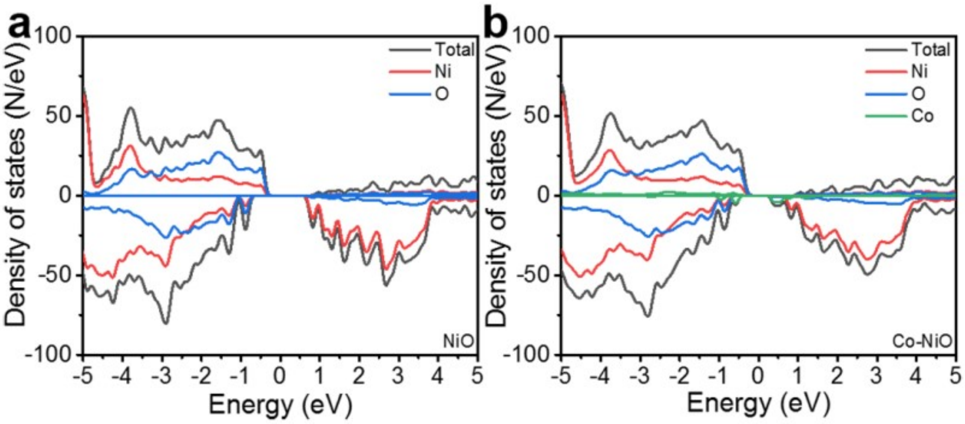

Fig. S14 The calculated DOS for (a) NiO and (b) Co doped NiO.

Fig. S14 shows partial DOS plots before and after Co substitution. Asymmetric DOS is observed due to magnetic Ni atoms. Pure Ni has a wide band gap of the electrons (0.80 eV) and has a semiconducting band structure, showing a semiconductor nature. After Co substitution, a significantly narrower band gap of 0.42 eV is observed for Co doped NiO, revealing the highly improved electronic conductivity of the Co-NiO/Fe_2O_3.

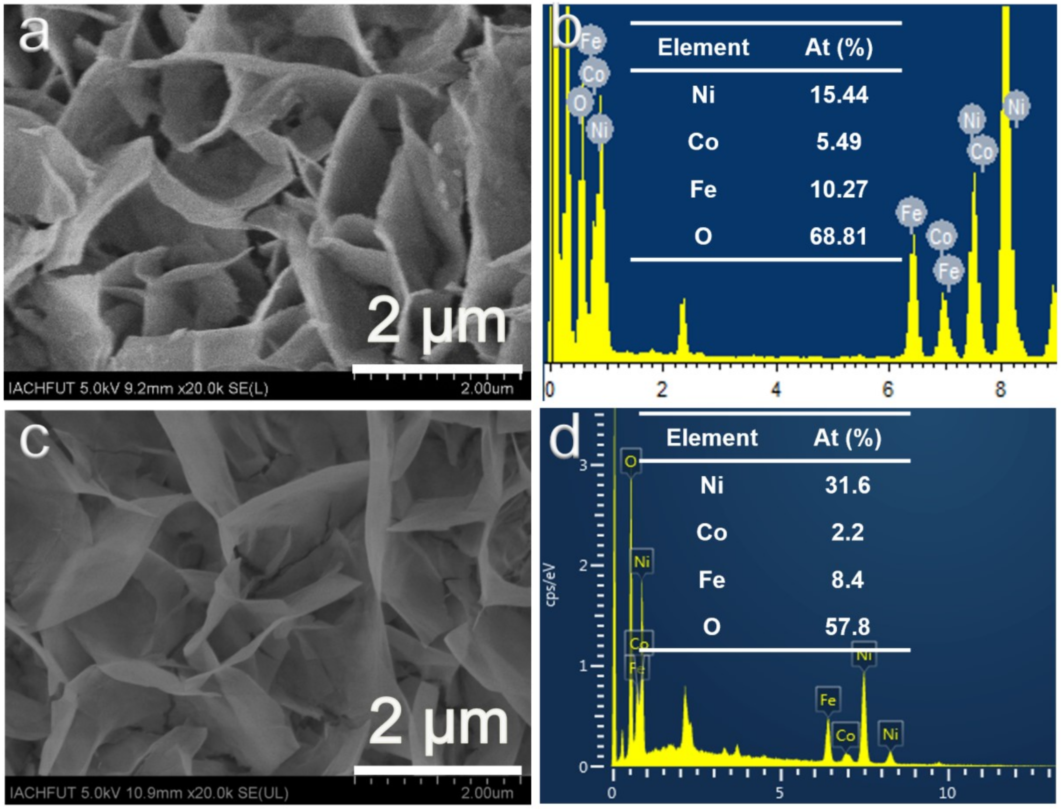

Fig. S15 FESEM images and EDS spectra of the Co-NiO/Fe_2O_3 before (a, b) and after (c, d) chronopotentiometry at 500 mA cm^{-2} for 300 hrs.

After long-term OER measurement, the morphology of Co-NiO/Fe_2O_3 remains the original nanosheet structure, revealing the high stability of the electrode. EDS spectrum of the Co-NiO/Fe_2O_3 after chronopotentiometry was further measured to investigate the reason for the slight stability degradation. As shown in Fig. S15d, EDS results confirm the presence of Ni, Co, and Fe after OER stability test. However, the atomic ratio of Co and Fe elements in the Co-NiO/Fe_2O_3 decreases to 2.2 and 8.4 at %, respectively. Co and Fe elements play an important role in the improvement of electrocatalytic performance of the Co-NiO/Fe_2O_3. The Co and Fe leaching during OER process may cause the slight stability degradation in a long-term stability test.

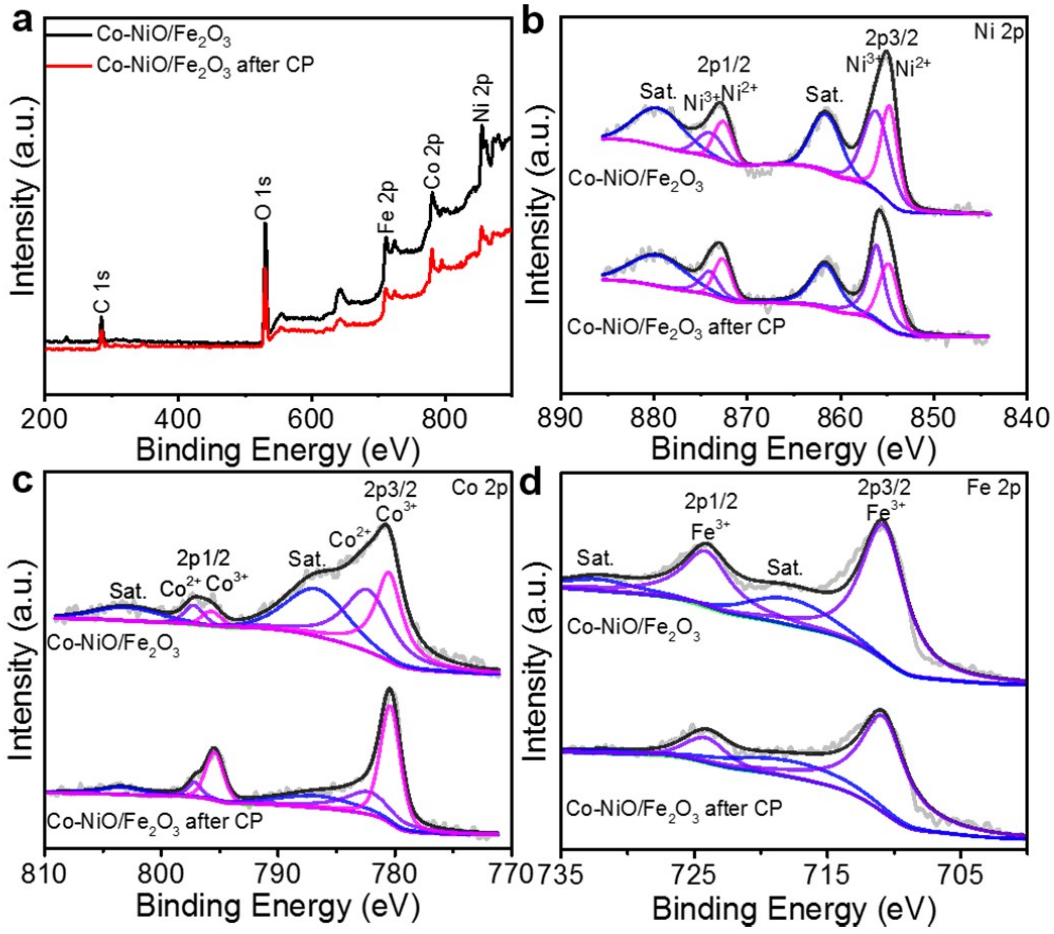

Fig. S16 XPS spectra of the Co-NiO/Fe_2O_3 before and after chronoamperometric operation. (a) survey, (b) Ni 2p, (c) Co 2p and (d) Fe 2p XPS spectra.

XPS spectra of the Co-NiO/Fe_2O_3 were collected to show the differences before and after stability test. Obviously, the results verify the presence of Ni, Co and Fe after OER stability test and show that the relative intensities of Ni^{3+} and Co^{3+} peaks are significantly increased, indicating that Ni and Co are oxidized to higher valence states which are considered as the real active sites in the previous reported results.

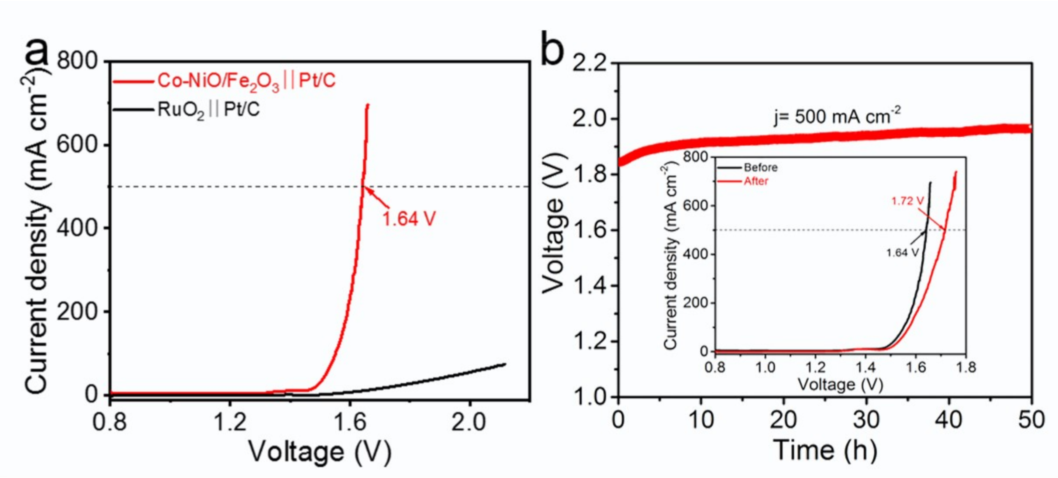

Fig. S17 Electrocatalytic activity for overall water splitting in 1.0 M KOH aqueous solution. (a) LSV polarization curves of the Co-NiO/Fe_2O_3 || commercial Pt/C cell and the commercial RuO_2 || Pt/C cell. (b) Long-term stability test of the Co-NiO/Fe_2O_3 || commercial Pt/C cell at constant current densities of 500 mA cm^{-2}.

The LSV polarization curve of the Co-NiO/Fe_2O_3 || Pt/C cell reveals that a low voltage of 1.64 V is required to reach 500 mA cm^{-2}, while the voltage of commercial RuO_2 || Pt/C cell is 1.65 V at a low current density of 10 mA cm^{-2} (Fig. S17a), suggesting the superior activity of the Co-NiO/Fe_2O_3 in overall water splitting. Furthermore, the Co-NiO/Fe_2O_3 || Pt/C cell exhibits high stability for electrolysis of water at 500 mA cm^{-2}, and the voltage of the cell only increases from 1.64 to 1.72 V after OER test for 50 hrs (Fig. S17b).

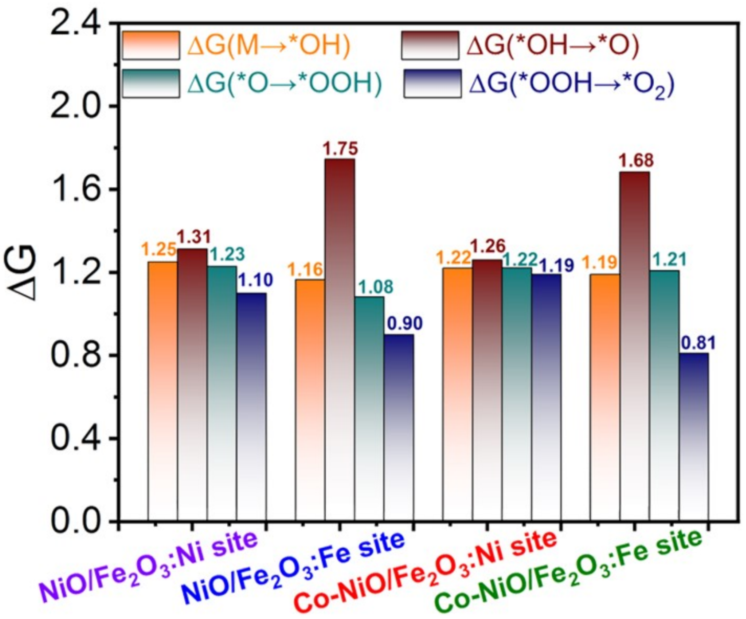

Fig. S18 *ΔG* of four elementary reactions ($M \rightarrow {}^{*}OH \rightarrow {}^{*}O \rightarrow {}^{*}OOH \rightarrow O_2$) for OER at Ni and Fe sites on surface model of NiO/Fe_2O_3 and $Co\text{-}NiO/Fe_2O_3$.

The elementary reaction with maximum change of *ΔG* is identified as rate-determining step (RDS) in the OER process. The second step from $^{*}OH$ to $^{*}O$ on the Ni and Fe sites in the NiO/Fe_2O_3 and $Co\text{-}NiO/Fe_2O_3$ has the lowest *ΔG* value and could be considered as RDS in our work. Co substitution in the $Co\text{-}NiO/Fe_2O_3$ reduces the energy barriers of RDS for both Ni (from 1.31 eV to 1.26 eV) and Fe (from 1.74 eV to 1.68 eV) sites, which contributes to the enhancement of OER activity.

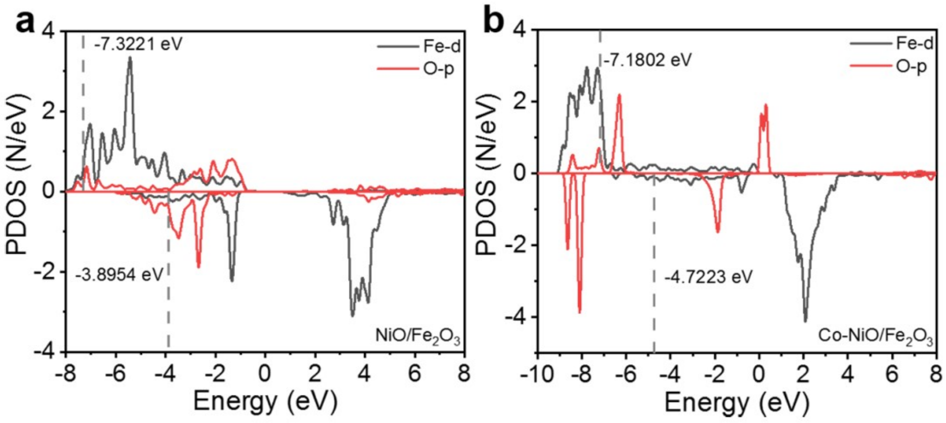

Fig. S19 Projected density of states (PDOS) of Fe *d* orbitals for (a) NiO/Fe_2O_3 and (b) Co-NiO/Fe_2O_3 at *OH intermediates.

The *d*-band center of Ni is -5.5427 eV and that of Fe is -7.1802 eV with respect to the Fermi energy level in the Co-NiO/Fe_2O_3. For the NiO/Fe_2O_3, the *d*-band center of Ni is -5.8353 eV and that of Fe is -7.3221 eV relative to the Fermi energy level. The *d*-band centers of Ni and Fe in the Co-NiO/Fe_2O_3 are closer to the Fermi energy level, implying the better OER activity after Co substitution.

Table S1. Lattice and structure parameters of the Co-NiO/Fe_2O_3 from XRD Rietveld refinement.

Phase No.		Fe_2O_3	Ni	Co-NiO
Space group		R-3c	Fm-3m	R-3m
Lattice parameters	a(Å)	5.0415(11)	3.52544	2.9556(20)
	b(Å)	5.0415(11)	3.52544	2.9556(20)
	c(Å)	13.7574(62)	3.52544	7.2271(13)

Table S2. Lattice and structure parameters of the NiO/Fe_2O_3 from XRD Rietveld refinement.

Phase No.		Fe_2O_3	Ni	NiO
Space group		R-3cH	Fm-3m	R-3mH
Lattice parameters	a(Å)	5.0489(23)	3.5238	2.950(15)
	b(Å)	5.0489(23)	3.5238	2.950(15)
	c(Å)	13.7568(77)	3.5238	7.240(75)

Table S3. OER performances of the Co-NiO/Fe_2O_3 compared with other advanced transition metal-based electrocatalysts in 1.0 M KOH alkaline solution.

Catalysts	Current density [mA cm^{-2}]	Overpotential [mV]	Tafel slope [mV dec^{-1}]	Stability	Reference
Co-NiO/Fe_2O_3	100<br>500	220<br>230	33.9	300h@500 mA cm^{-2}	This work
$NiCo_{2-x}Fe_xO_4$	10<br>100	274<br>~320	42	25h@10 mA cm^{-2}	7
CeO_{2-x}–FeNi	10	195	43	48h@10 mA cm^{-2}	8
$FeCoSeO_x$	10	294	45.1	40h@10 mA cm^{-2}	9
Cu@CeO_2@NiFeCo-0.25	10	230	32.7	30h@10 mA cm^{-2}	10
Fe–Co–O/Co@NC-mNS/NF	10	257	41.56	50h@10 mA cm^{-2}	11
$CoNiFeO_x$-NC	100	286	64.05	40h@1.49V	12
$NiFe_2O_{4-x}$/NMO-25	100	304	42.7	40 h@200 mA cm^{-2}	13
CoNi/$CoFe_2O_4$/NF	100	290	45	48 h@100 mA cm^{-2}	14
Ir-NiO	10	215	38	10 h@10 mA cm^{-2}	15
Fe-NiO-Ni CHNAs	10	245	43.4	24 h@10 mA cm^{-2}	16
Co_3O_4/CoO	10	302	68.6	4*10^4 s@10 mA cm^{-2}	17
CoVFeN@NF	10<br>100	212<br>264	34.8	50h@100 mA cm^{-2}	18
$(Ni_xFe_{1-x})_2P$	10<br>100	166<br>~250	59.3	24h@10 mA cm^{-2}<br>24h@100 mA cm^{-2}	19
Cu@NiFe LDH	10<br>100	199<br>281	27.8	48h@10 mA cm^{-2}<br>48h@100 mA cm^{-2}	20
Ni_5Co_3Mo-OH	100	304	56.4	100h@100 mA cm^{-2}	21
Ni_3S_2/MnO_2	10<br>100	260<br>348	61	48 h@100 mA cm^{-2}	22
e-ICLDH@GDY/NF	100	249	43.6	80 h@100 mA cm^{-2}	23
S-doped (Ni,Fe)OOH	100	281	48.9	100 h@100 mA cm^{-2}	24
$NiMoO_x$/NiMoS	100	225	34	25 h@100 mA cm^{-2}	25

Table S4. The EIS fitting data of different catalysts at 1.47 V (vs RHE).

Sample	R_s[Ω]	R_{ct}[Ω]	CPE_1[F]
Co-NiO/Fe_2O_3	1.028	1.015	0.5262
NiO	3.078	7.818	0.6797
Fe_2O_3	1.618	5.834	0.7246
NiO/Fe_2O_3	1.642	1.823	0.5580

References

1. P. Giannozzi, S. Baroni, N. Bonini, M. Calandra, R. Car, C. Cavazzoni, D. Ceresoli, G. L. Chiarotti, M. Cococcioni, I. Dabo, A. D. Corso, S. Gironcoli, S. Fabris, G. Fratesi, R. Gebauer, U. Gerstmann, C. Gougoussis, A. Kokalj, M. Lazzeri, L. M. Samos, N. Marzari, F. Mauri, R. Mazzarello, S. Paolini, A. Pasquarello, L. Paulatto, C. Sbraccia, S. Scandolo, G. Sclauzero, A. P. Seitsonen, A. Smogunov, P. Umari and R. M. Wentzcovitch, *J. Phys.:Condens. Matter*, 2009, **21**, 395502.
2. P. Giannozzi, O. Andreussi, T. Brumme, O. Bunau, M. B. Nardelli, M. Calandra, R. Car, C. Cavazzoni, D. Ceresoli, M. Cococcioni, N. Colonna, I. Carnimeo, A. D. Corso, S. Gironcoli, P. Delugas, R. A. DiStasio Jr, A. Ferretti, A. Floris, G. Fratesi, G. Fugallo, R. Gebauer, U. Gerstmann, F. Giustino, T. Gorni, J. Jia, M. Kawamura, H. Y. Ko, A. Kokalj, E. Küçükbenli, M. Lazzeri, M. Marsili, N. Marzari, F. Mauri, N. L. Nguyen, H. V. Nguyen, A. Otero-de-la-Roza, L. Paulatto, S. Poncé, D. Rocca, R. Sabatini, B. Santra, M. Schlipf, A. P. Seitsonen, A. Smogunov, I. Timrov, T. Thonhauser, P. Umari, N. Vast, X. Wu and S. Baroni, *J.Phys.:Condens.Matter*, 2017, **29**, 465901.
3. J. P. Perdew, K. Burke and M. Ernzerhof, *Phys. Rev. Lett.*, 1996, **77**, 3865−3868.
4. P. E. Blöchl, O. Jepsen and O. K. Andersen, *Phys. Rev. B*, 1994, **49**, 16223−16233.
5. G. Kresse and D. Joubert, *Phys. Rev. B*, 1999, **59**, 1758−1775.
6. H. J. Monkhorst and J. D. Pack, *Phys. Rev. B*, 1976, **13**, 5188−5192.
7. Y. Huang, S. Zhang, X. Lu, Z. Wu, D. Luan and X. Lou, *Angew. Chem. Int. Ed.*, 2021, **60**, 11841–11846.
8. J. Yu, J. Wang, X. Long, L. Chen, Q. Cao, J. Wang, C. Qiu, J. Lim and S. Yang, *Adv. Energy Mater.*, 2020, **11**, 2002731.
9. Q. Qian, Y. Li, Y. Liu and G. Zhang, *Appl. Catal. B: Environ.*, 2020, **266**, 118642.
10. J. Xia, H. Zhao, B. Huang, L. Xu, M. Luo, J. Wang, F. Luo, Y. Du and C. Yan, *Adv. Funct. Mater.*, 2020, **30**, 1908367.
11. T. I. Singh, G. Rajeshkhanna, U. N. Pan, T. Kshetri, H. Lin, N. H. Kim and J. H. Lee, *Small*, 2021, **17**, 2101312.
12. C. Chen, Y. Tuo, Q. Lu, H. Lu, S. Zhang, Y. Zhou, J. Zhang, Z. Liu, Z. Kang, X. Feng, D. Chen, *Appl. Catal. B: Environ.*, 2021, **287**, 119953.
13. J. Choia, D. Kim, W. Zheng, B. Yan, Y. Li, L. Yoon, S. Lee and Y. Piao, *Appl. Catal. B:*

Environ., 2021, **286**, 119857.

14. S. Li, S. Sirisomboonchai, A. Yoshida, X. An, X. Hao, A. Abudula and G. Guan, *J. Mater. Chem. A*, 2018, **6**, 19221–19230.
15. Q. Wang, X. Huang, Z. Zhao, M. Wang, B. Xiang, J. Li, Z. Feng, H. Xu and M. Gu, *J. Am. Chem. Soc.*, 2020, **142**, 7425–7433.
16. Y. Lei, T. Xu, S. Ye, L. Zheng, P. Liao, W. Xiong, J. Hu, Y. Wang, J. Wang, X. Ren, C. He, Q. Zhang, J. Liu and X. Sun, *Appl. Catal. B: Environ.*, 2021, **285**, 119809.
17. Z. Liu, Z. Xiao, G. Luo, R. Chen, C. Dong, X. Chen, J. Cen, H. Yang, Y. Wang, D. Su, Y. Li and S. Wang, *Small*, 2019, **15**, 1904903.
18. D. Liu, H. Ai, J. Li, M. Fang, M. Chen, D. Liu, X. Du, P. Zhou, F. Li, K. Lo, Y. Tang, S. Chen, L. Wang, G. Xing and H. Pan, *Adv. Energy Mater.*, 2020, **10**, 2002464.
19. S. Sun, X. Zhou, B. Cong, W. Hong and G. Chen, *ACS Catal.*, 2020, **10**, 9086−9097.
20. L. Yu, H. Zhou, J. Sun, F. Qin, F. Yu, J. Bao, Y. Yu, S. Chen and Z. Ren, *Energy Environ. Sci.*, 2017, **10**, 1820−1827.
21. S. Hao, L. Chen, C. Yu, B. Yang, Z. Li, Y. Hou, L. Lei and X. Zhang, *ACS Energy Lett.*, 2019, **4**, 952−959.
22. Y. Xiong, L. Xu, C. Jin and Q. Sun, *Appl.Catal. B: Environ.*, 2019, **254**, 329−338.
23. L. Hui, Y. Xue, B. Huang, H. Yu, C. Zhang, D. Zhang, D. Jia, Y. Zhao, Y. Li, H. Liu and Y. Li, *Nat. Commun.*, 2018, **9**, 5309.
24. L. Yu, L. Wu, B. McElhenny, S. Song, D. Luo, F. Zhang, Y. Yu, S. Chen and Z. Ren, *Energy Environ. Sci.*, 2020, **13**, 3439−3446.
25. P. Zhai, Y. Zhang, Y. Wu, J. Gao, B. Zhang, S. Cao, Y. Zhang, Z. Li, L. Sun and J. Hou, *Nat. Commun.*, 2020, **11**, 5462.
